# Efficacy and safety of Jinlida granules as an adjuvant treatment for diabetic nephropathy: a systematic review and meta-analysis

**DOI:** 10.3389/fendo.2026.1740623

**Published:** 2026-02-03

**Authors:** Bo Dai, Yanxu Chen, Yang Xiao, Jinying Chen, Zexin Zhu, Peng Zhang, Jieyu Zhang, Jian Sun, Pengjie Bao, Zheng Nan, Qi Zhang

**Affiliations:** 1 Changchun University of Chinese Medicine, Changchun, China; 2 College of Science and Technology Changchun, Changchun, China; 3 The Affiliated Hospital of Changchun University of Chinese Medicine, Changchun, China; 4 Shenzhen Hospital (Futian) of Guangzhou University of Chinese Medicine, Shenzhen, China

**Keywords:** diabetic nephropathy, Jinlida granules, meta-analysis, randomized controlled trials, system review, traditional Chinese medicine

## Abstract

**Objective:**

Jinlida Granules (JLD), a patented traditional Chinese medicine, has demonstrated significant efficacy when used as an adjunct treatment for DN. This meta-analysis systematically evaluated the efficacy, safety, and renoprotective effects of JLD as adjunctive treatment in DN patients.

**Methods:**

We systematically searched the Chinese literature databases (China National Knowledge Infrastructure, Wanfang Data, and China Science and Technology Journal Database) and English literature databases (PubMed, Web of Science, Cochrane Library, and Embase) from inception to September 2025 and included relevant studies published in Chinese and English after screening according to predefined inclusion and exclusion criteria. Meta-analysis and bias assessment of the included studies were conducted using Stata 16 and Review Manager 5.4.1 software. The quality of included studies was evaluated using the risk-of-bias tools outlined in the *Cochrane Handbook*.

**Results:**

This study analyzed data from 13 randomized controlled trials (RCTs) with 1,333 participants, including 658 participants in the control group and 675 participants in the treatment group. As an adjunctive therapy for DN, JLD treatment enhances clinical efficacy rate [RR = 1.30 (95% CI: 1.21, 1.39), I² =27%] and reduces the levels of serum creatinine (SCr) [SMD = -2.01 (95% CI: -2.33, -1.69), I² =80.0%], blood urea nitrogen (BUN) [SMD = -0.79 (95% CI: -1.07, -0.52), I² = 80%], 24-h urine protein test (24h-UTP) [SMD = -1.44 (95% CI: -1.88, -1.00), I² =89%], urinary albumin excretion rates (UAER) [SMD = -2.14 (95% CI: -2.97, -1.30), I² =92%], fasting plasma glucose (FPG) [SMD = -0.63 (95% CI: -1.01, -0.24), I² =83%], 2-h plasma glucose (2hPG) [SMD = -0.71 (95% CI: -1.20, 0.23), I² =89%], hemoglobin A1c (HbA1c) [SMD = -0.95 (95% CI: -1.55, -0.35), I² =92%], total cholesterol (TC) [SMD = -0.91 (95% CI: -1.75, -0.08), I² =92%], triglycerides (TG) [SMD = -3.07 (95% CI: -6.06, -0.08), I² =99%], vascular endothelial growth factor (VEGF) [SMD = -1.50 (95% CI: -2.53, -0.48), I² =95%], IGF-1 [SMD = -0.59 (95% CI: -0.97, -0.21), I² =74%], IL-6 [SMD = -1.77 (95% CI: -2.46, -1.09), I² =81%], TNF-α [SMD = -1.75 (95% CI: -2.37, -1.13), I² =88%], and high sensitivity C-reactive protein (hs-CRP) [SMD = -2.48 (95% CI: -2.81, -2.15), I² =54%]. For adverse reactions, the pooled risk ratio was 0.71 (95% CI: 0.42, 1.20, I² = 0%) in the JLD adjunct therapy group relative to controls. The 95% CI crossing 1 indicated no statistically significant difference in adverse reaction rates, and no reliable conclusion regarding the safety superiority of JLD could be drawn.

**Conclusions:**

JLD as an adjunctive therapy enhances renal function, glucose and lipid metabolism, inflammatory regulation, and vascular health in patients with DN. Meta-analysis revealed no statistically significant differences in safety outcomes, indicating that safety remains a matter of debate.

**Systematic Review Registration:**

https://www.crd.york.ac.uk/PROSPERO/view/CRD420251148725, identifier CRD420251148725

## 1 Introduction

Diabetic nephropathy (DN) is a prevalent microvascular complication of diabetes mellitus (DM) and represents one of the principal causes of end-stage renal disease (ESRD). Clinical manifestations of DN include proteinuria, thickening of the glomerular basement membrane, and progressive tubulointerstitial fibrosis. The pathogenesis of DN involves dysregulated glucose metabolism, hemodynamic disturbances, and enhanced oxidative stress (1). Although modern medical interventions can relieve a few symptoms of DN, they are associated with low clinical efficacy and significant side effects (2). The global burden of DN is increasing constantly because of the lack of effective interventions. In 2021, the global prevalence of DN was 107.6 million, which is a 5.1% decrease since 1990. However, deaths from the disease reached 477,300 worldwide that same year, marking a 37.8% increase since 1990 (3). Currently, the standard treatment for DN patients includes hypoglycemic therapy, blood pressure management, lipid regulation, and microcirculation improvements (4). DN management also involves comprehensive lifestyle modifications such as restricted protein intake, smoking cessation, and limited salt intake, as well as pharmacological interventions such as oral intake of SGLT-2 inhibitors, GLP-1 receptor agonists, and statins (5, 6). Advances in medical technology as well as antioxidant and anti-inflammatory therapies have also shown promising therapeutic benefits for DN patients. However, these medications cannot be used to specifically treat DN because their side effects may exacerbate patient discomfort (7).

The international recognition for traditional Chinese medicine (TCM) has steadily increased in recent years. TCM has also demonstrated distinct advantages in the treatment of DN (8). JinLiDa Granules (JLD), a Chinese patent medicine for treating DN (9), produced by Shijiazhuang Yiling Pharmaceutical Co., Ltd., contains 17 herbal ingredients, including *Ginseng (Panax ginseng C. A.Mey.), Rehmannia glutinosa, Cornus officinalis (Cornus officinalis Sieb. et Zucc), Polyporia cocos, Eupatorium fortunei (Eupatorium fortunei Turcz.), Rhizoma coptidis (Coptis chinensis Franch.), Rhizoma anemarrhenae (Anemarrhena asphodgfoides), Salvia miltiorrhiza (Salvia miltiorrhiza Bge.), Pachyrhizua angulatus (Pueraria thomsonii Benth.), Cortex lycii radices (Cortex Lycii), Polygonati rhizoma (Polygonatum sibiricum), Sophora flavescens (Sophora flavescens Ait), Radix ophiopogonis (Ophiopogon japonicus), Semen Litchi*, *Polygonum multiflorum (Polygonum multiflorum Thunb.), Epimedium (Herba epimedil)*, and *Atractylodes lancea preparata (Atractylodes* sp*ecies)*. The composition of JLD and its preparation method are shown in **Supplementary Materials S1**, **2**. We followed established guidelines for standardizing the scientific nomenclature of botanical drug components in JLD and validated their names through cross-referencing with “The World Flora Online” (http://www.worldfloraonline.org). JLD exhibits minimal side effects while effectively delaying or reversing disease progression. Previous reports also demonstrate that JLD is effectively improves blood glucose levels, proteinuria, and renal function (10).

Although multiple Meta-analyses have examined the application of TCM in treating DN, a systematic review and meta-analysis on JLD as an adjunctive therapy remains necessary for two primary reasons. First, numerous high-quality randomized controlled trials (RCTs) have recently been published on the use of JLD in DN treatment. These studies confirm that JLD effectively lowers blood glucose levels in diabetic patients (11). Animal studies also confirm JLD’s efficacy in alleviating DN with favorable safety profiles (12). Second, although multiple RCTs have demonstrated JLD’s ability to mitigate DN progression, no dedicated Meta-analysis on this drug’s treatment of DN has been reported. The absence of a comprehensive quantitative assessment of existing evidence prevents clear determination of its actual clinical value for DN management. Therefore, this study systematically integrates existing clinical evidence to comprehensively evaluate the efficacy and safety of JLD as an adjunctive therapy for DN through meta-analysis, aiming to provide evidence-based guidance for clinical practice.

## 2 Methods

This meta-analysis and systematic review follows the PRISMA guidelines and is registered with the International Prospective Register of Systematic Reviews (PROSPERO) (Registration No. CRD420251148725). The PRISMA 2020 checklist is shown in **Supplementary Material S3**.

### 2.1 Database and search strategy

The literature search for this study was conducted in both Chinese and English-language databases from inception to September 2025 and was restricted to studies published in Chinese and English. The search included Chinese databases, including China National Knowledge Infrastructure, Wanfang Data, and the China Science and Technology Journal Database, as well as the English databases, including PubMed, Embase, the Cochrane Library, and Web of Science. Furthermore, we manually searched potential sources, including grey literature. The primary search terms were “diabetic nephropathy,” “Jinlida Granules,” “Jinlida,” “randomized controlled trial,” and “RCT.” The Search strategies are detailed in **Supplementary Material S4**.

### 2.2 Study selection criteria

The key characteristics of the study selection criteria were defined according to the PICOS framework and included five key components of the trials: Participants, Interventions, Comparators, Outcomes, and Study designs.

#### 2.2.1 Participants

This study included all RCTs targeting DN patients without restrictions on the patient age, disease duration, ethnicity, or geographic location.

#### 2.2.2 Interventions

Patients in the treatment group received JLD in addition to the standard treatment regimen used for the control group. The pharmaceutical product known as JLD was manufactured by Shijiazhuang Yiling Pharmaceutical Co., Ltd. The national drug approval number is Z20050845. The specification of JLD is 9 g per sachet (finished product weight), and each sachet corresponds to a total crude drug dosage of 45 g (detailed crude drug composition of the 17 herbal ingredients is provided in **Supplementary Material S1**, in accordance with the Chinese Pharmacopoeia (2020 Edition, Volume I) and official technical data from the manufacturer). The administration method was as follows: one sachet was dissolved in hot water for oral administration, three times daily (tid), with an 8-week course of treatment. The dosage of the finished product is calculated at 0.45 g·kg^-1^·d^-1^, and that of the crude drug is 2.25 g·kg^-1^·d^-1^. These values are based on a commonly used average body weight of 60 kg for adult patients in clinical studies. The total dose for the 8-week course is 1512 g for the finished product and 7560 g for the crude drug.

#### 2.2.3 Comparators

The treatment regimen for the control group in this study was designed to provide comprehensive management, which incorporated symptom control, diabetes education, dietary modifications, exercise interventions, and blood glucose monitoring. Oral hypoglycemic therapy focused on selecting drugs with minimal nephrotoxicity, including metformin, SGLT-2 inhibitors, thiazolidinediones, and α-glucosidase inhibitors. Angiotensin-converting enzyme inhibitors (ACEIs) and angiotensin receptor blockers (ARBs) were used for antihypertensive therapy. Lipid-lowering therapy involved the use of fibrates.

#### 2.2.4 Outcomes

There were 3 distinct outcome measures in this study: primary, secondary, and safety outcome measures.

Primary outcome measures: The primary outcomes consisted of the clinical efficacy rate, serum creatinine (Scr), blood urea nitrogen (BUN), and 24-hour urine protein quantification (24h-UTP). These measures were used to assess clinical improvements in the renal function of patients with DN.

Secondary outcome measures: The secondary outcomes included fasting blood glucose (FBG), 2-hour postprandial glucose (2hPG), glycated hemoglobin (HbA1c), total cholesterol (TC), triglycerides (TG), vascular endothelial growth factor (VEGF), insulin-like growth factor-1 (IGF-1), interleukin-6 (IL-6), tumor necrosis factor-α (TNF-α), and high-sensitivity C-reactive protein (hs-CRP). These measures were used to comprehensively evaluate the therapeutic effects of JLD on blood glucose control, lipid profiles, inflammatory markers, and relevant growth factors.

Safety outcome measures: The safety profile of treatment regimens was evaluated by documenting all adverse events occurring during RCTs, including hypoglycemic reactions, gastrointestinal discomfort, and other drug-related adverse reactions.

#### 2.2.5 Study design

All included studies in this study were RCTs focused on patients with DN.

### 2.3 Exclusion criteria

#### 2.3.1 Type of case report

Animal studies, case reports, meta-analyses, reviews, conference proceedings, and letters were excluded.

#### 2.3.2 Types of participants

Patients receiving kidney dialysis and those with ambiguous or unclear diagnostic criteria were excluded from this study.

#### 2.3.3 Interventions

Other forms of TCM such as acupuncture, cupping, and acupressure patch therapy were excluded from this study.

#### 2.3.4 Outcome measures

Studies with incomplete or missing results were excluded from this study.

### 2.4 Literature screening and data extraction

Two researchers (Yanxu Chen and Jinying Chen) created a literature database in the EndNote X9 software and extracted the required research data. They used a cross-validation method to verify the inclusion and exclusion criteria for the data. In case of disagreements, a third researcher (Bo Dai) was invited to participate in the adjudication and a final decision was reached through mutual consultation.

### 2.5 Risk of bias assessment

Quality of the included studies was independently assessed by Jieyu Zhang and Jian Xu using the Cochrane Risk of Bias tool and cross-verified. In the case of a disagreement, two researchers (Shilin Liu and Zexin Zhu) discussed the findings and reached a consensus.

### 2.6 Statistical analysis

Statistical data analysis was performed using Review Manager 5.4.1 and Stata 16 software. For dichotomous outcome measures, relative risk (RR) and 95% confidence interval (CI) were reported as effect sizes. For continuous outcome measures, if studies employed identical measurement tools and units, mean difference (MD) and 95% CI were reported. If measurement tools or units differed, standardized mean difference (SMD) and 95% CI were used to combine effect sizes.

Heterogeneity was assessed using a combined Q-test and I² statistic, with the I² value and its 95% CI reported to quantify the degree of heterogeneity and estimate uncertainty. The P-value threshold for the Q-test was set at 0.1, a traditional standard for meta-analyses. This threshold is justified because clinical studies of traditional Chinese medicine are susceptible to factors such as syndrome differentiation, combination with basic treatment regimens, and varying treatment durations, leading to potentially high heterogeneity. Setting the P-value threshold at 0.1 enhances sensitivity in identifying heterogeneity, balances Type I and Type II errors, and prevents overlooking critical sources of heterogeneity. Regarding interpretation of the I² statistic, we strictly adhere to the gradient description outlined in the 7th edition of the Cochrane Handbook for Systematic Reviews of Interventions: 0%–40% indicates low or no apparent heterogeneity; 30%–60% suggests moderate heterogeneity; 50%–90% indicates substantial heterogeneity; 75%–100% indicates considerable heterogeneity. The model selection strategy is as follows: if Q test P > 0.1 and I² is 0%–40%, indicating low heterogeneity, use a fixed-effects model to pool effect sizes; if Q test P < 0.1 or I² ≥ 50%, investigate sources of heterogeneity through subgroup analysis and use a random-effects model to pool effect sizes. Sensitivity analysis assessed the stability of results by sequentially removing and then recombining effect sizes after inclusion in the study. Publication bias was evaluated using funnel plots combined with Egger’s test.

### 2.7 GRADE assessment of included studies

The 13 included studies were evaluated using the GRADE approach and their quality was graded as high, moderate, low, or very low. The specific assessments included risk of bias, consistency, indirectness, precision, and publication bias. Risk of bias was assessed through random sequence generation, allocation concealment, blinding of investigators and participants, completeness of outcome data, and selective reporting. Consistency refers to the degree of agreement between different outcome measures or subgroup analyses, whereas applicability evaluates the alignment of study subjects, interventions, and outcomes with the target population or clinical question. Precision was assessed based on sample size, effect size magnitude, and the width of the 95% CI. Publication bias was determined by evaluating the tendency to publish positive results.

## 3 Results

### 3.1 Literature screening results

**Figure 1:**
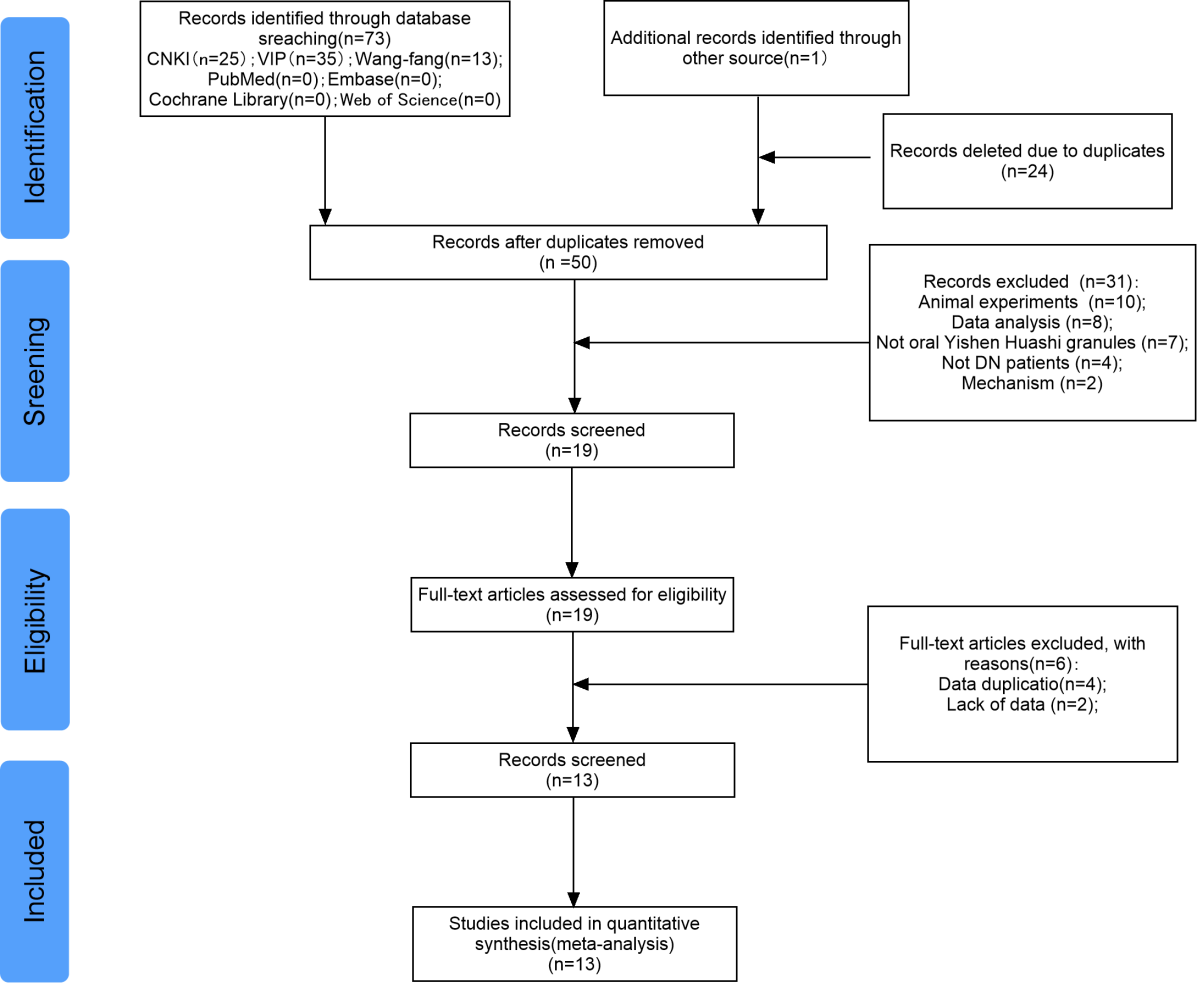
Flowchart of study selection strategy.

The literature screening process is shown in **Figure 1**. This study initially retrieved 74 studies. Then, 24 duplicate studies were excluded. Furthermore, after assessing the titles and abstracts, 31 studies were excluded. Subsequently, 4 duplicate studies and 1 study with missing data were excluded. Finally, 13 studies were included in this meta-analysis (13–25).

### 3.2 Basic information of the included studies

Details of the included studies are shown in **Table 1**. This study included 13 RCTs published in Chinese between 2012 and 2025. The sample sizes varied from 60 to 150 participants. Overall, the study consisted of 1,333 patients, including 675 in the treatment group and 658 in the control group.

**Table 1:** Baseline characteristics for inclusion.

The basic characteristics of the included literatures												
Document source	Sample size	Male/female/case		Average age		Average course of disease		Intervention	Control	Course of treatment per wk	Outcome index	Adverse reaction
	T/C	T/C		T/C		T/C						
Chen Jianshou 2019	41/41	25/17	27/15	51.2 ± 2.0	50.70 ± 2.5	10.8 ± 2.4	10.6 ± 2.6	JLD+Tongxinluo	Irbesartan	12	⑤⑥⑦⑱	Report
Liu Hongli 2019	60/60	37/23	34/26	64.32 ± 8.11	65.85± 8.56	5.04 ± 2.59	4.83 ± 3.12	JLD+Tongxinluo	Benazepril	12	①②③④⑤⑧⑩⑪⑫⑬	Report
Yu Gengxu 2016	50/50	35/15	23/27	46.7	47.1	Unclear		JLD	CT	4	⑤⑥⑦⑭⑮	NO Report
Su Yan 2017	75/75	40/35	45/30	53.8 ± 6.2	54.5 ± 5.6	Unclear		JLD	CT	4	①⑤⑥⑦⑮	NO Report
Ren Xiao 2019	53/53	29/24	30/23	52.40 ± 4.30	52.50 ± 4.50	3.80 ± 0.60	3.80 ± 0.50	JLD	CT	12	①⑤⑥⑦⑧⑭⑮	Report
Xu Bo 2018	64/64	37/27	33/31	45.78 + 3.76	45.80 + 3.92	12.31 + 2.02	11.97 + 2.06	JLD+Tongxinluo	Irbesartan	12	①②③④⑤⑥⑦⑰⑱	Report
Du Yanxia 2012	56/55	33/23	35/20	53 ± 8	52 ± 9	Unclear		JLD+Tongxinluo	Irbesartan	12	⑤⑥⑦	Report
Deng Chengzong 2020	30/30	17/13	15/15	55.11 ± 7.95	54.97 ± 7.86	7.11 ± 0.58	7.08 ± 0.49	JLD+Liraglutide	Irbesartan	12	②③④⑥⑦⑯⑰⑱	Report
Liu Qiang 2024	55/47	30/25	25/22	58.05 ± 5.75	57.85 ± 5.75	5.21 ± 1.20	5.17 ± 1.19	JLD+Tripterygium Tablet	Tripterygium Tablet	8	①⑤⑥⑦⑧⑯⑰⑱	Report
Dai Hongsha 2023	55/47	31/24	26/21	47.36 ± 5.41	46.73 ± 5.29	10.87 ± 1.65	11.06 ± 1.89	JLD+Irbesartan	Irbesartan	8	①②③④⑤⑥⑦⑨⑰⑱	Report
Xia Meiling 2025	45/45	28/17	26/19	59.7 ± 4.9	59.8 ± 4.9	12.54 ± 2.30	12.15 ± 2.11	JLD+Dapagliflozin	Dapagliflozin	12	①②③④⑤⑥⑩⑪⑯⑰⑱	Report
Wang Junhua 2023	44/44	26/18	23/21	66.01 ± 5.12	65.90 ± 4.01	1.97 ± 0.56	2.10 ± 0.75	JLD+Benazepril	Benazepril	12	①②③⑤⑥⑦⑭	NO Report
Su Jie 2020	4747	53/41		62.83 ± 7.29		8.79 ± 2.33		JLD+Benazepril	Benazepril	12	②③④⑧⑩⑪⑭⑮	Report

①, Clinically effective; ②, FBG; ③, 2h PG; ④, HbA1c; ⑤, Scr; ⑥, BUN; ⑦, 24h UTP; ⑧, UAcr; ⑨, eGFR; ⑩, TC; ⑪, TG; ⑫, LDL; ⑬, HDL; ⑭, VEGF; ⑮, IGF-1; ⑯, IL-6; ⑰, TNF-α; ⑱, hs-CRP.

CT, conventional therapy; JLD, Jinlida Granules.

### 3.3 Risk of bias assessment

**Figure 2:**
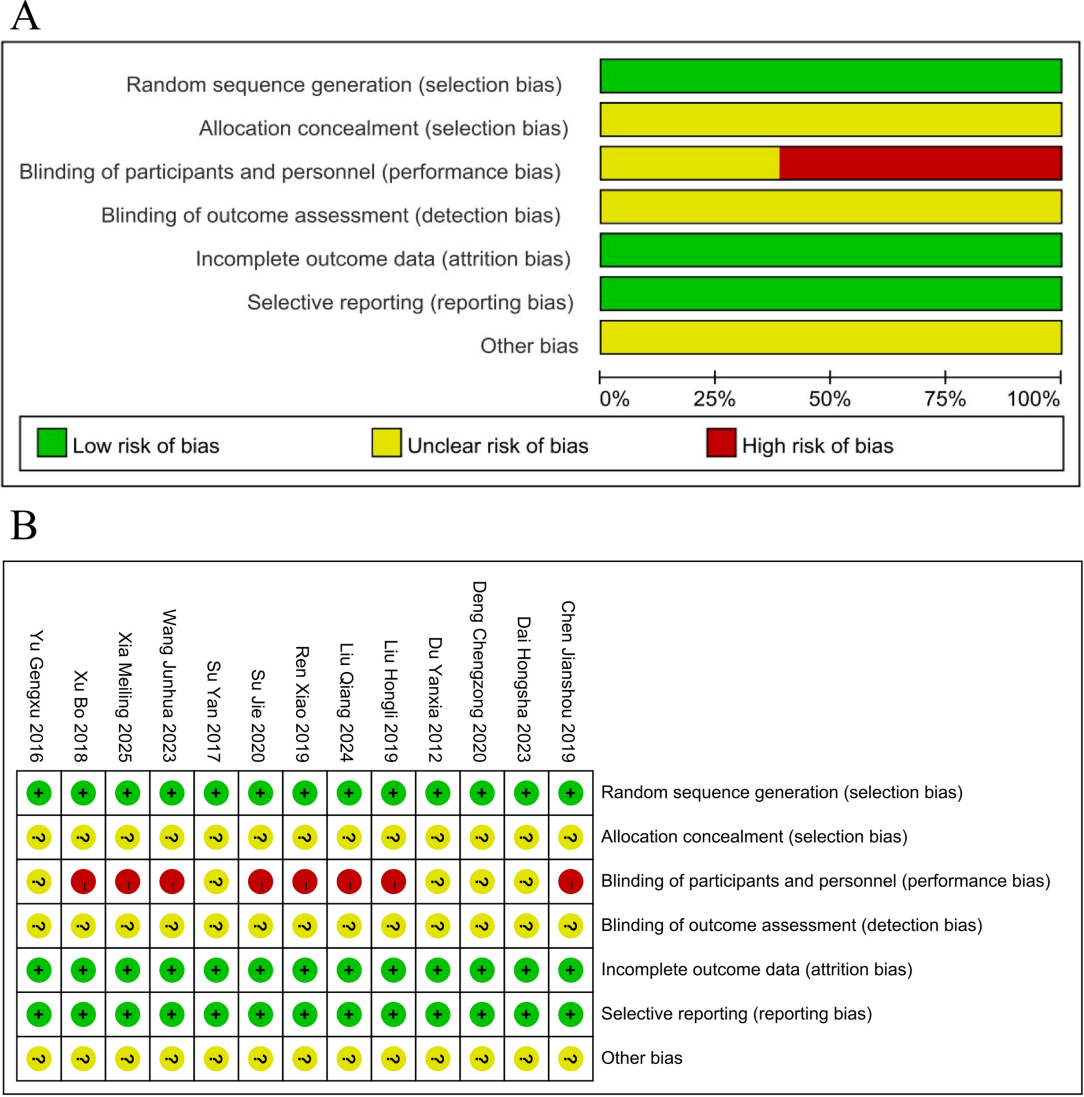
**(A)** Risk of bias graph. **(B)** Risk of bias summary.

Details of the risk of bias ratings are shown in **Figure 2**. All the 13 studies were rated as “low risk” for the use of random sequence generation. All 13 studies were rated as “unclear” for allocation concealment because of inadequate descriptions. Five studies were rated as “unclear” for failing to specify double-blinding status, whereas the remaining 8 were rated as “high risk” for not evaluating blinding. All studies were categorized as “low risk” because of the availability of complete data and lack of selective reporting. Other types of bias were not mentioned across studies and were rated as “unclear.”

### 3.4 Meta-analysis results for primary outcomes

#### 3.4.1 Clinical effectiveness rate

**Figure 3:**
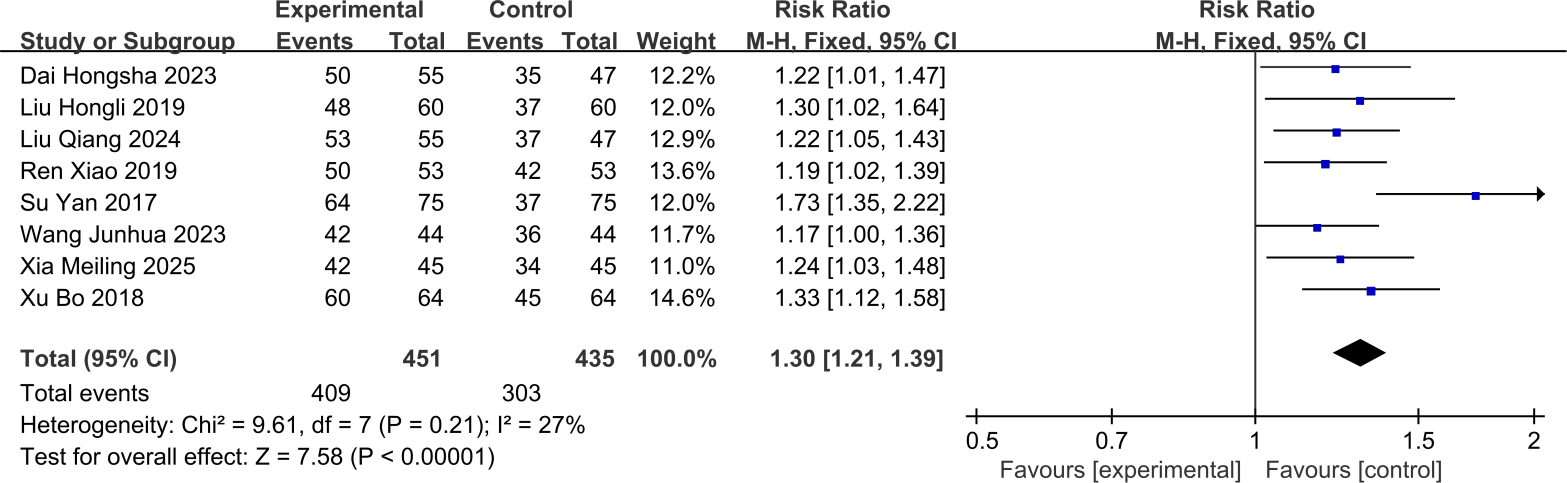
Forest plot of the Clinical effectiveness rate.

The clinical efficacy rate in this study included 8 RCTs with 886 patients (treatment group = 451 patients; control group = 435 patients). These 8 studies showed low heterogeneity (I² = 27%, P > 0.1). Therefore, a fixed-effects model was used to pool the effect sizes. The treatment group showed significantly superior clinical efficacy compared to the control group [RR = 1.30 (95% CI: 1.21, 1.39), P < 0.001]. The forest plot for clinical efficacy rate is shown in **Figure 3**.

#### 3.4.2 SCr

**Figure 4:**
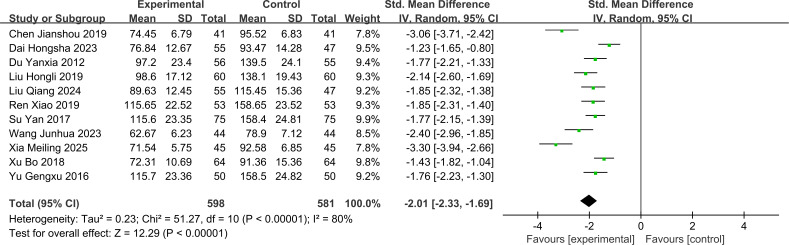
Forest plot for Serum creatinine levels.

The SCr analysis in this study included 11 RCTs with 1,179 patients (treatment group = 598 patients; control group = 581 patients). Since substantial heterogeneity was observed between the 11 studies (I² = 80%, P < 0.1), a random-effects model was used to pool the effect sizes. The SCr levels were significantly lower in the treatment group compared to the control group [SMD = -2.01 (95% CI: -2.33, -1.69), P < 0.001]. The forest plot for SCr is shown in **Figure 4**.

The subgroup analysis by intervention strategy showed that JLD monotherapy [SMD = -1.79 (95% CI: -2.04, -1.54)], JLD combined with Tongxinluo [SMD = -2.06 (95% CI: -2.66, -1.46)], and JLD combined with other conventional interventions [SMD = -2.29 (95% CI: -3.50, -1.09)] all significantly reduced SCr levels. Although numerically superior efficacy was observed in the combination therapy subgroups compared to monotherapy, the difference between subgroups was not statistically significant (Chi²=1.22, df=2, P = 0.54, I²=0%), which may be due to insufficient statistical power caused by the small sample size in the “JLD combined with other conventional interventions” subgroup. Stratified analysis by intervention duration revealed significantly superior outcomes in the >8 weeks subgroup [SMD = -2.24 (95% CI: -2.71, -1.77)] compared to the ≤8 weeks subgroup [SMD = -1.65 (95% CI: -1.93, -1.37)], suggesting that extending the JLD treatment cycle can sustainably reduce serum creatinine levels. The differences between subgroups were statistically significant (Chi²=4.53, df=1, P = 0.03, I²=77.9%). For stratification by disease duration, the treatment group demonstrated superior efficacy in the ≤10 years disease duration subgroup [SMD = -1.95 (95% CI: -2.22, -1.69)] compared to the >10 years disease duration subgroup [SMD = -1.44 (95% CI: -1.67, -1.22)], suggesting that JLD exhibits greater restorative potential for early renal impairment. Statistically significant differences were observed between the subgroups (Chi²=8.31, df=1, P = 0.004, I²=88%). Additionally, stratified by the control group’s background therapy, the JLD-containing experimental group significantly reduced SCr in both subgroups: the RAAS Blocker subgroup [SMD = -1.97 (95% CI: -2.46, -1.49), P < 0.001] and the Non-RAAS Blocker subgroup [SMD = -2.06 (95% CI: -2.52, -1.61), P < 0.001]. Notably, there was no statistically significant difference in SCr-lowering efficacy between these two subgroups (Chi²=0.07, df=1, P = 0.79, I²=0%), suggesting that the SCr-lowering efficacy of JLD is consistent regardless of whether the control group uses RAAS blockers or other background therapies. The forest plot for this subgroup analysis of SCr is presented in **Supplementary Material S5**.

#### 3.4.3 BUN

**Figure 5:**
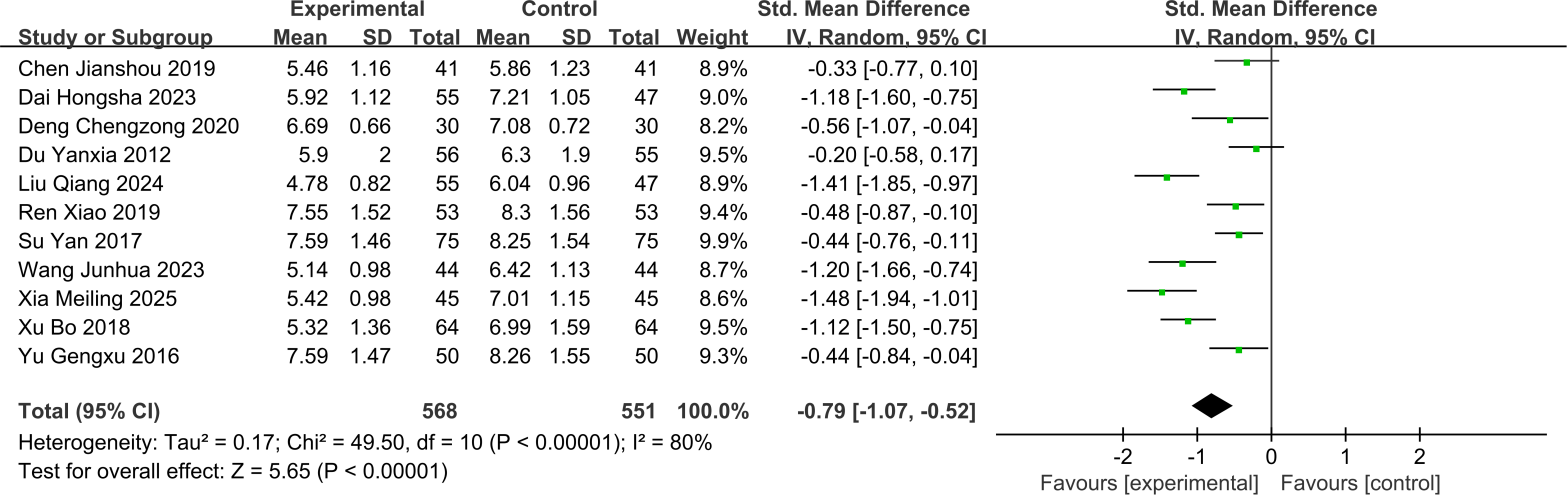
Forest plot for Blood urea nitrogen levels.

The BUN analysis in this study included 11 RCTs with 1,119 patients (treatment group = 568 patients; control group = 551 patients). Since substantial heterogeneity was observed among the 11 studies (I² = 80%, P < 0.1), a random-effects model was used to pool the effect sizes. The treatment group showed significantly lower BUN levels compared to the control group [SMD = -0.79 (95% CI: -1.07, -0.52), P < 0.001]. The forest plot is shown in **Figure 5**.

The subgroup analysis by intervention strategy showed that JLD monotherapy [SMD = -0.45 (95% CI: -0.66, -0.24)], JLD combined with Tongxinluo [SMD = -0.53 (95% CI: -1.04, -0.01)], and JLD combined with other conventional interventions [SMD = -1.12 (95% CI: -1.47, -0.76)] all significantly reduced BUN levels. JLD combination therapy exhibited superior efficacy over monotherapy, and the difference between subgroups was statistically significant (Chi²=10.15, df=2, P = 0.006, I²=80.3%), indicating a synergistic effect of JLD combined with other interventions on BUN reduction. Stratified analysis by intervention duration revealed numerically better BUN-lowering outcomes in the ≤8 weeks subgroup [SMD = -0.85 (95% CI: -1.34, -0.37)] than in the >8 weeks subgroup [SMD = -0.76 (95% CI: -1.13, -0.40)]. This suggests JLD exerts a rapid regulatory effect on urea metabolism in the early phase, which subsequently stabilizes with prolonged treatment. Notably, the difference between subgroups was not statistically significant (Chi²=0.09, df=1, P = 0.77, I²=0%), possibly due to the small sample size in the stratified subgroups leading to insufficient statistical power. For stratification by disease duration, patients with disease duration >10 years [SMD = -1.02 (95% CI: -1.48, -0.57)] showed numerically superior therapeutic efficacy compared with those with disease duration ≤10 years [SMD = -0.82 (95% CI: -1.42, -0.22)], suggesting JLD may exert stronger regulatory effects on glomerular urea permeability in patients with long-term diabetic nephropathy (DN), thereby achieving more pronounced BUN reduction. However, no statistically significant difference was observed across subgroups (Chi²=0.29, df=1, P = 0.59, I²=0%). Additionally, stratified by the control group’s background therapy, the JLD-containing experimental group significantly reduced BUN in both subgroups: the RAAS Blocker subgroup [SMD = -0.77 (95% CI: -1.14, -0.39), P < 0.01] and the Non-RAAS Blocker subgroup [SMD = -0.83 (95% CI: -1.28, -0.38), P = 0.0003]. Notably, there was no statistically significant difference in BUN-lowering efficacy between these two subgroups (Chi²=0.05, df=1, P = 0.82, I²=0%), suggesting that the BUN-lowering efficacy of JLD is consistent regardless of whether the control group uses RAAS blockers or other background therapies.The forest plot for subgroup analyses of BUN is shown in **Supplementary Material S6**.

#### 3.4.4 24h-UTP

**Figure 6:**
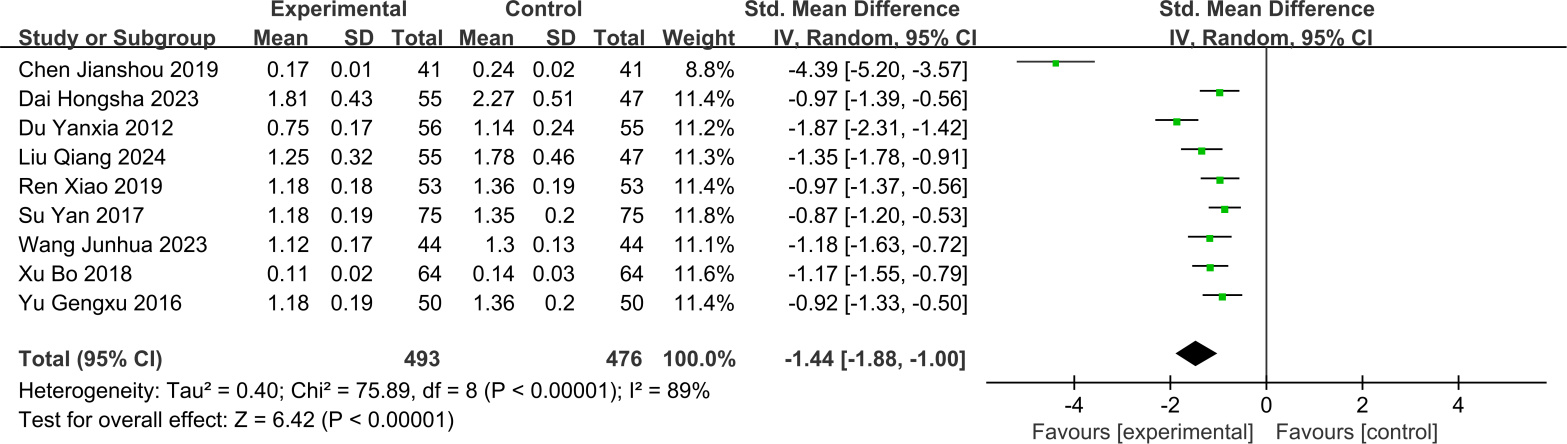
Forest plot for 24-hour urine protein quantification.

The 24h-UTP analysis in this study included 9 RCTs with 969 patients (treatment group = 493 patients; control group = 476 patients). Since substantial heterogeneity was observed between the 9 studies (I² = 89%, P < 0.1), a random-effects model was used to pool the effect sizes. The 24h-UTP levels were significantly lower in the treatment group compared to the control group [SMD = -1.44 (95% CI: -1.88, -1.00), P < 0.001]. The forest plot for 24h-UTP is shown in **Figure 6**.

The subgroup analysis further indicated that JLD monotherapy [SMD = -0.91 (95% CI: -1.13, -0.69)], JLD combined with Tongxinluo [SMD = -2.42 (95% CI: -3.88, -0.97)], and JLD combined with other conventional interventions [SMD = -1.07 (95% CI: -1.37, -0.76)] all significantly reduced 24h-UTP levels. Although JLD combination therapy was numerically superior to monotherapy, no statistically significant difference was observed across subgroups (Chi²=4.48, df=2, P = 0.11, I²=55.3%), which may be attributed to moderate heterogeneity among subgroups and insufficient statistical power. Stratified analysis by intervention duration showed that patients with treatment duration >8 weeks [SMD = -1.85 (95% CI: -2.67, -1.03)] had significantly better 24h-UTP-lowering outcomes than those with duration ≤8 weeks [SMD = -1.00 (95% CI: -1.21, -0.80)], suggesting that extending the JLD treatment cycle can sustainably reduce 24h-UTP levels. The difference between subgroups was marginally statistically significant (Chi²=3.84, df=1, P = 0.05, I²=73.9%). For stratification by disease duration, patients with disease duration >10 years [SMD = -2.12 (95% CI: -3.64, -0.61)] showed numerically superior efficacy compared with those with disease duration ≤10 years [SMD = -1.15 (95% CI: -1.52, -0.77)]. However, no statistically significant difference was observed between subgroups (Chi²=1.51, df=1, P = 0.22, I²=33.8%), implying that JLD may exert stronger regulatory effects on glomerular protein permeability in patients with long-term DN, thereby achieving more pronounced 24h-UTP reduction. Additionally, stratified by the control group’s background therapy, the JLD-containing experimental group significantly reduced 24h-UTP in both subgroups: the RAAS Blocker subgroup [SMD = -1.85 (95% CI: -2.67, -1.03), P < 0.001] and the Non-RAAS Blocker subgroup [SMD = -1.00 (95% CI: -1.20, -0.80), P < 0.001]. Notably, there was a marginally statistically significant difference in 24h-UTP-lowering efficacy between these two subgroups (Chi²=4.22, df=1, P = 0.05, I²=74.2%), suggesting that the 24h-UTP-lowering efficacy of JLD may have a slight difference depending on whether the control group uses RAAS blockers or other background therapies. The forest plot for subgroup analyses of 24h-UTP is shown in **Supplementary Material S7**.

### 3.5 Secondary and security outcomes

This study assessed 12 secondary outcome measures, including glucose metabolism, lipid metabolism, inflammatory factors, and growth factors, as well as security outcomes associated with adverse events. Meta-analysis data for these outcomes are shown in **Table 2**, and the forest plots for these secondary outcomes are shown in **Supplementary Materials S8–S19**.

**Table 2:** Secondary outcomes and safety outcomes.

Outcome measure	Included in the literature	Quantity	N (T/C)	Testing for heterogeneity		Meta-analysis		
				I^2^/%	P	SMD	95% CI	
UAER	Liu Hongli 2019, Liu Qiang 2024, Ren xiao 2019, Su Jie 2020	4	215/207	92%	<0.001	-2.14	[-2.97, -1.30]	
FBG	Dai Hongsha 2023, Deng Chengzong 2020, Liu Hongli 2019, Su Yan 2017, Wang Junhua 2023, Xia Meiling 2025, Xu Bo 2018	7	345/337	83%	0.001	-0.63	[-1.01,-0.24]	
2hPG	Dai Hongsha 2023, Deng Chengzong 2020, Liu Hongli 2019, Su Yan 2017, Wang Junhua 2023, Xia Meiling 2025, Xu Bo 2018	7	345/337	89%	0.004	-0.71	[-1.20,0.23]	
HbA1c	Dai Hongsha 2023, Deng Chengzong 2020, Liu Hongli 2019, Su Jie 2020, Xia Meiling 2025, Xu Bo 2018	6	301/293	92%	0.002	-0.95	[-1.55,-0.35]	
TC	Liu Hongli 2019, Su Jie 2020, Xia Meiling 2025	3	152/152	92%	0.03	-0.91	[-1.75,-0.08]	
TG	Liu Hongli 2019, Su Jie 2020, Xia Meiling 2025	3	152/152	99%	0.04	-3.07	[-6.06,-0.08]	
IGF-1	Ren Xiao 2019, Su Jie 2020, Su Yan 2017, Yu Gengxu 2016	4	225/225	74%	0.002	-0.59	[-0.97,-0.21]	
VEGF	Ren Xiao 2019, Su Jie 2020, Wang Junhua 2023, Yu Gengxu 2016	4	194/194	95%	0.004	-1.50	[-2.53,-0.48]	
hs-CRP	Chen Jianshou 2019, Dai Hongsha 2023, Deng Chengzong 2020, Liu Qiang 2024, Xia Meiling 2025, Xu Bo 2018	6	290/274	54%	<0.001	-2.48	[-2.81,-2.15]	
IL-6	Deng Chengzong 2020, Liu Qiang 2024, Xia Meiling 2025	3	130/122	81%	<0.001	-1.77	[-2.46,-1.09]	
TNF-α	Dai Hongsha 2023, Deng Chengzong 2020, Liu Qiang 2024, Xia Meiling 2025, Xu Bo 2018	5	249/233	88%	<0.001	-1.75	[-2.37,-1.13]	
Adverse effect	Chen Jianshou 2019, Dai Hongsha 2023, Deng Chengzong 2020, Du Yanxia 2012, Liu Hongli 2019, Liu Qiang 2024, Ren Xiao 2019, Su Jie 2020, Xia Meiling 2025, Xu Bo 2018	10	506/489	0%	0.2	0.71	[0.42,1.20]	

#### 3.5.1 Secondary outcome

Secondary outcomes were categorized into four domains (glucose metabolism, lipid metabolism, inflammatory factors, and growth factors) for analysis. Given the moderate to high heterogeneity observed across studies, random-effects models were consistently applied to pool effect sizes to account for inter-study variability. Detailed results are presented below:

For glucose metabolism assessment using FBG, 2hPG, and HbA1c, combined JLD therapy significantly reduced FBG levels [SMD = -0.63 (95% CI: -1.01, -0.24), I² = 83%], 2hPG levels [SMD = -0.71 (95% CI: -1.20, -0.23), I² = 89%], and HbA1c levels [SMD = -0.95 (95% CI: -1.55, -0.35), I² = 92%] in DN patients. In terms of lipid-lowering effects, combined JLD therapy significantly decreased TC levels [SMD = -0.91 (95% CI: -1.75, -0.08), I² = 92%], but no statistically significant reduction in TG was observed [SMD = -3.07 (95% CI: -6.06, 0.08), I² = 99%], as the 95% CI crossed 0. Regarding anti-inflammatory activity measured by TNF-α, hs-CRP, and IL-6, combined JLD therapy led to a marked reduction in TNF-α [SMD = -1.75 (95% CI: -2.37, -1.13), I² = 88%], hs-CRP [SMD = -2.48 (95% CI: -2.81, -2.15), I² = 54%], and IL-6 [SMD = -1.77 (95% CI: -2.46, -1.09), I² = 81%] levels. Additionally, analysis of growth factors (VEGF and IGF-1) showed that combined JLD therapy significantly downregulated VEGF [SMD = -1.50 (95% CI: -2.53, -0.48), I² = 95%] and IGF-1 [SMD = -0.59 (95% CI: -0.97, -0.21), I² = 74%] expression levels.

Overall, combined JLD therapy exerted significant beneficial effects on glucose metabolism regulation, inflammation suppression, and pro-fibrotic growth factor downregulation in DN patients, whereas its lipid-lowering efficacy was only evident for TC reduction.

#### 3.5.2 Security outcome

Safety data were collected from 13 RCTs included in this meta-analysis, involving 1,333 participants (675 in the JLD combined therapy group and 658 in the control group). Among these studies, 10 explicitly reported adverse events (AEs), with a total of 31 mild AEs documented in **Table 3**. The overall incidence of AEs in the JLD group ranged from 3.64% to 10.00%, predominantly characterized by mild gastrointestinal reactions (62.5%, n=19/31), including abdominal distension, gastric discomfort, nausea, and mild diarrhea. Other infrequent AEs included hypoglycemia (12.5%, n=4/31), rash (10.0%, n=3/31), dizziness/headache (7.5%, n=2/31), and orthostatic hypotension (7.5%, n=2/31). Notably, no severe AEs (e.g., hepatotoxicity, nephrotoxicity, myelosuppression) were reported across all studies. Meta-analysis of AE rates showed no statistically significant difference between the JLD group and the control group [RR = 0.71 (95% CI: 0.42, 1.20), I² = 0%), indicating that JLD adjunctive therapy does not increase additional safety risks compared to conventional treatment alone. Most AEs were self-limiting or resolved spontaneously without specific interventions, further supporting the mild and reversible nature of JLD-related adverse reactions in clinical settings.

**Table 3:** Detailed summary table of adverse reactions.

Literature identification	Type of adverse reaction	Number of cases with adverse reactions	Total number of cases	Incidence rate (kept to 2 decimal places)	Management method
Ren Xiao 2019	Treatment group: Abdominal distension; Control group: Nausea and vomiting	Treatment group: 1 case; Control group: 1 case	Treatment group: 53 cases; Control group: 53 cases	Treatment group: 1.89%; Control group: 1.89%	All symptoms were mild, resolved spontaneously without any intervention
Xu Bo 2018	Treatment group: Gastric discomfort; Control group: Postural hypotension, rash	Treatment group: 1 case; Control group: 3 cases (1 case of hypotension + 2 cases of rash)	Treatment group: 64 cases; Control group: 64 cases	Treatment group: 1.56%; Control group: 4.69%	Treatment group: Medication taken after meals, symptoms disappeared; Control group: Symptoms disappeared after symptomatic treatment
Du Yanxia 2012	Treatment group: Gastric discomfort; Control group: Dyspepsia, mild diarrhea, postural hypotension	Treatment group: 2 cases; Control group: 4 cases (1 case of dyspepsia + 2 cases of diarrhea + 1 case of hypotension)	Treatment group: 56 cases; Control group: 55 cases	Treatment group: 3.57%; Control group: 7.27%	Treatment group: Medication taken after meals, symptoms disappeared; Control group: Specific intervention not clearly stated, presumed mild reactions resolved spontaneously
Deng Chengzong 2020	Treatment group: Hypoglycemia, diarrhea; Control group: Hypoglycemia, diarrhea, hypotension, headache; Study group: Hypoglycemia, diarrhea, headache	Treatment group: 3 cases (1 case of hypoglycemia + 1 case of diarrhea + 1 case of headache); Control group: 4 cases (1 case of hypoglycemia + 1 case of diarrhea + 1 case of hypotension + 1 case of headache)	Treatment group: 30 cases; Control group: 30 cases;	Treatment group: 10.00%; Control group: 13.33%	Specific management method not clearly stated; literatures mentioned all adverse reactions were mild, presumed to resolve spontaneously or with symptomatic treatment
Liu Qiang 2024	Treatment group: Dry mouth, fatigue, nausea, rash; Control group: Dry mouth, fatigue, nausea	Treatment group: 5 cases (1 case of dry mouth + 1 case of fatigue + 1 case of nausea + 2 cases of rash); Control group: 4 cases (1 case of dry mouth + 1 case of fatigue + 2 cases of nausea)	Treatment group: 55 cases; Control group: 47 cases	Treatment group: 9.09%; Control group: 8.51%	Specific management method not clearly stated; literatures indicated no statistically significant difference in the incidence of adverse reactions, presumed mild reactions relieved with symptomatic treatment
Dai Hongsha 2023	Treatment group: Gastric discomfort, nausea and vomiting; Control group: Nausea and vomiting, gastric discomfort	Treatment group: 2 cases (1 case of gastric discomfort + 1 case of nausea and vomiting); Control group: 3 cases (1 case of nausea and vomiting + 2 cases of gastric discomfort)	Treatment group: 55 cases; Control group: 47 cases	Treatment group: 3.64%; Control group: 6.38%	Specific management method not clearly stated, presumed mild gastrointestinal reactions resolved spontaneously or with symptomatic intervention
Xia Meiling 2025	Treatment group: Hypoglycemia, decreased appetite, skin itching; Control group: Hypoglycemia, decreased appetite	Treatment group: 4 cases (1 case of hypoglycemia + 2 cases of decreased appetite + 1 case of skin itching); Control group: 2 cases (1 case of hypoglycemia + 1 case of decreased appetite)	Treatment group: 45 cases; Control group: 45 cases	Treatment group: 8.89%; Control group: 4.44%	Specific management method not clearly stated; literatures mentioned no statistically significant difference in adverse reactions, presumed mild reactions resolved spontaneously
Su Jie 2020	Treatment group: Flatulence, nausea; Control group: Flatulence, dizziness, headache, nausea	Treatment group: 2 cases (1 case of flatulence + 1 case of nausea); Control group: 5 cases (1 case of flatulence + 2 cases of dizziness + 1 case of headache + 1 case of nausea)	Treatment group: 47 cases; Control group: 47 cases	Treatment group: 4.26%; Control group: 10.64%	Specific management method not clearly stated, presumed mild reactions relieved with symptomatic treatment or disappeared spontaneously
Chen Jianshou 2019	Treatment group: Epigastric discomfort; Control group: Dyspepsia, postural hypotension, mild diarrhea	Treatment group: 2 cases; Control group: 3 cases (1 case of dyspepsia + 1 case of postural hypotension + 1 case of mild diarrhea)	Treatment group: 41 cases; Control group: 41 cases	Treatment group: 4.88%; Control group: 7.32%	Treatment group: Medication taken after meals, symptoms resolved spontaneously; Control group: Specific intervention not clearly stated, presumed mild reactions improved spontaneously
Liu Hongli 2019	Treatment group: Epigastric discomfort; Control group: Abdominal distension, decreased appetite	Treatment group: 1 case; Control group: 2 cases (1 case of abdominal distension + 1 case of decreased appetite)	Treatment group: 60 cases; Control group: 60 cases	Treatment group 1.67%; Control group: 3.33%	Specific management method not clearly stated, presumed mild gastrointestinal reactions resolved spontaneously

### 3.6 Publication bias analysis

**Figure 7:**
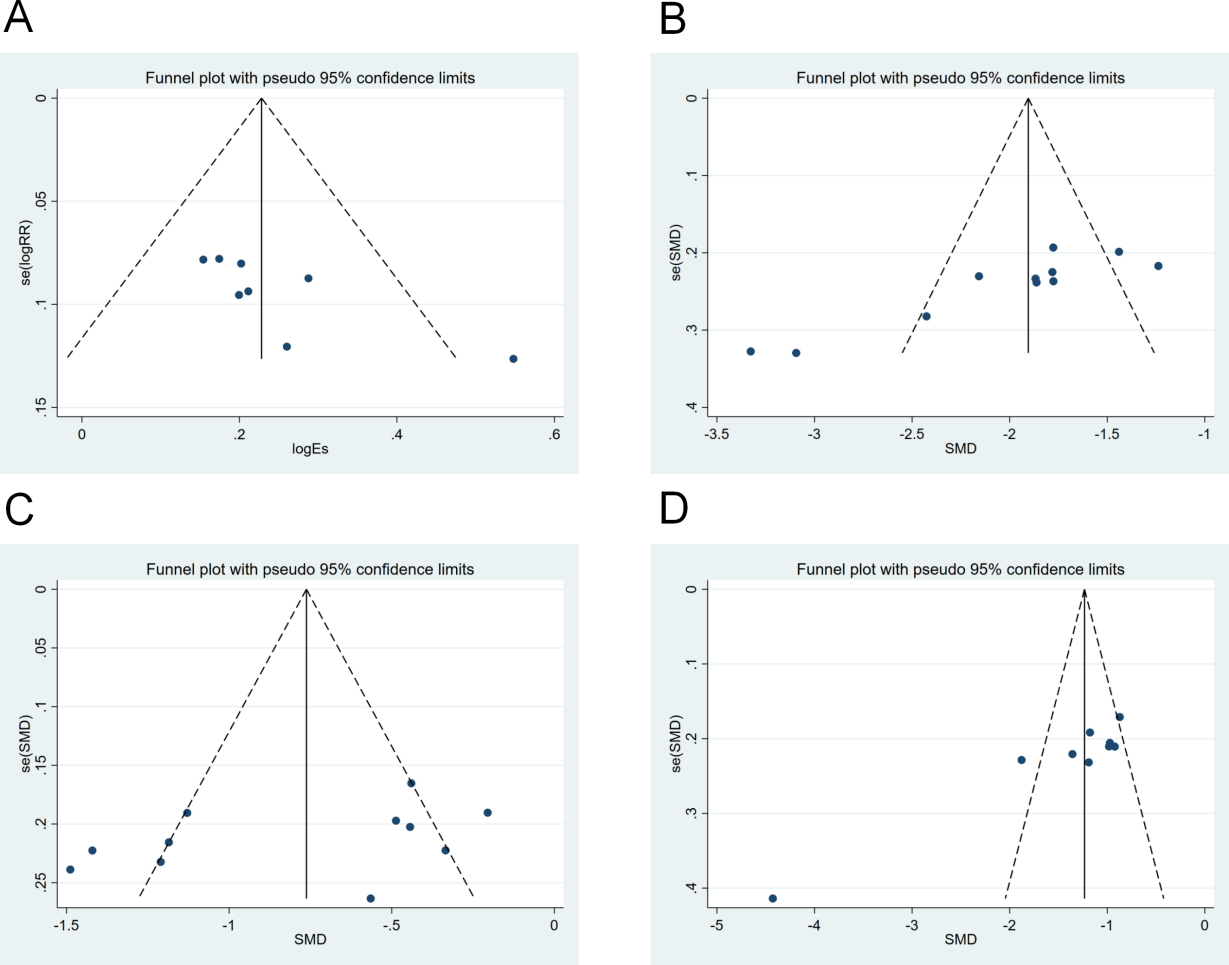
Funnel plots for **(A)** Clinical effectiveness rate, **(B)** Serum creatinine, **(C)** Blood urea nitrogen, and **(D)** 24-hour urine protein quantification.

This study assessed publication bias for primary outcome measures (including clinical response rate, SCr, BUN, and 24h-UTP) using an inverted funnel plot (**Figures 7A–D**). Publication bias was comprehensively assessed by combining visual inspection of the funnel plot with Egger’s linear regression test. Outcomes with potential publication bias were further quantitatively corrected using the trim-and-fill method. 8 studies reporting clinical response rate (**Figure 7A**): Visual funnel plot inspection revealed asymmetric scatter distribution with fewer and sparsely distributed points on the right side, and points not uniformly distributed around the centerline. Combined with the Egger test (P = 0.026 < 0.10), this indicated potential publication bias. 11 studies reporting SCr (**Figure 7B**): The funnel plot visually revealed a unilateral clustering trend, with points concentrated on the left and a noticeable absence on the right. The Egger test result (P = 0.000 < 0.10) further confirmed significant publication bias. 11 studies reporting BUN (**Figure 7C**): Visual assessment of the funnel plot showed data points distributed approximately symmetrically around the pooled effect size, with balanced numbers and densities on both sides and no obvious unilateral gaps. The Egger test (P = 0.171 > 0.10) also indicated no significant publication bias. 9 studies reporting 24h-UTP (**Figure 7D**): Visual inspection of the funnel plot revealed asymmetric scatter distribution, with sparse points on the left and clustered points on the right, indicating significant one-sided attrition. The Egger test (P = 0.001 < 0.10) suggested potential publication bias. After performing trim-and-fill correction for the biased outcome measures (clinical effectiveness rate, SCr, 24h-UTP), no significant missing studies were identified. The adjusted pooled effect size showed no statistically significant difference from the original pooled effect size, indicating that the meta-analysis results were minimally affected by publication bias and are robust.

### 3.7 Sensitivity analysis

This sensitivity analysis for the primary outcome measures showed high heterogeneity (**Supplementary Materials S20–S22****).** After excluding some studies, the overall heterogeneity was reduced. However, some results still demonstrate significant heterogeneity. Further exclusion of the remaining studies did not significantly alter the results.

### 3.8 GRADE assessment of evidence quality

GRADE evaluation showed that all 13 studies included in this study were RCTs focused on the treatment of DN with JLD. According to the GRADE grading system, 12 studies showed medium quality of evidence and 1 study showed low quality of evidence. The results for the quality of evidence regarding JLD combined therapy for DN were as follows (1): evidence level for patient response rates from 8 studies was rated as moderate, thereby indicating high credibility of the research findings; (2) evidence level for effect on SCr from 11 studies was rated as low because of a high risk of bias and significant heterogeneity between studies; (3) evidence level for effect on BUN from 11 studies was rated as low, thereby indicating limited reliability of the findings; (4) evidence level for effect on 24h-UTP from 9 studies was low, thereby suggesting that the JLD combination therapy had low efficacy in reducing 24h-UTP in DN patients. However, significant heterogeneity between studies and presence of the risk of bias in some studies limited the reliability of the conclusions. The GRADE evidence for the main outcome indicators is shown in **Table 4**.

**Table 4:** The GRADE evidence of the main outcome indicators.

		Certainty assessment					No. of patients		Effect	Certainty	Importance
Outcome indicator (No. of studies)	Study design	Risk of bias	Inconsistency	Indirectness	Imprecision	Other considerations	JLD+CT	CT	Absolute (95% CI)		
Clinical effectiveness rate (8)	Randomized trials	Moderate	Low	Low	Low	None	451	435	RR = 1.30, 95% CI (1.21, 1.39), I² =27%	⨁⨁⨁◯ Moderate	IMPORTANT
SCR (11)	Randomized trials	Moderate	serious	Moderate	Moderate	None	598	581	SMD = -2.01, 95% CI (-2.33, -1.69), I² =80%	⨁⨁◯◯ Low	IMPORTANT
BUN (11)	Randomized trials	Moderate	serious	Moderate	Low	None	568	551	SMD = -0.79, 95% CI (-1.07, -0.52), I² = 80%	⨁⨁◯◯ Low	IMPORTANT
24h-UTP (9)	Randomized trials	Moderate	serious	Moderate	Moderate	None	493	476	SMD = -1.44, 95% CI (-1.88, -1.00), I² =89%	⨁⨁◯◯ Low	IMPORTANT

## 4 Discussion

The pathogenesis of DN is multifactorial demonstrating synergy between inflammatory responses, oxidative stress imbalance, and metabolic dysregulation. Hyperglycemia is a primary initiating factor that induces characteristic pathological changes such as glomerulosclerosis and tubulointerstitial fibrosis by activating oxidative stress, facilitating the accumulation of advanced glycation end-products (AGEs), and impairing renal hemodynamics. Among the immune factors, macrophages, T- and B-cells, and inflammatory mediators such as including TNF-α and IL-6 significantly exacerbate renal inflammation and fibrosis (26). Metabolic dysregulation involving insulin resistance and impaired insulin signaling exacerbates renal injury through multiple signaling pathways, including PI3K/Akt, mTOR, Wnt/β-catenin, JAK/STAT, and NF-κB (27). Furthermore, cross-regulation and a positive feedback loop between inflammation and metabolic abnormalities accelerates the progression of DN. Since DN pathogenesis is multifactorial and synergistic, the multi-targeted and multi-pathway therapeutic approach of TCM has shown clear clinical value as an adjunct or alternative treatment option in the management of DN (28). This includes improved renal function and delayed disease progression. Current research studies have focused on the identification of bioactive components in TCM and systematic elucidation of their molecular mechanisms for renal protection. TCM exerts effects by modulating specific signaling pathways, including alleviating the dysregulation of glucose and lipid metabolism, inhibiting oxidative stress damage, reducing renal inflammatory responses, delaying the progression of renal interstitial and glomerular fibrosis, and preserving structural and functional integrity of the podocytes. These pathways have been confirmed as key targets for TCM intervention in DN (8). Therefore, to broaden the therapeutic options for DN, this study analyzed RCTs focusing on JLD to validate the efficacy and safety of JLD in the treatment of DN.

### 4.1 Mechanism of action of JLD

TCM posits that the core pathogenesis of DN lies in qi and yin deficiency coupled with spleen-kidney insufficiency. JLD, formulated from a rationally designed combination of 17 Chinese herbs, precisely adheres to the core therapeutic principle of “tonifying qi and nourishing yin, strengthening the spleen and benefiting the kidneys.” From a modern medical perspective, DN is a multi-pathway pathological disorder involving renal inflammatory responses, glomerulosclerosis, renal interstitial fibrosis, oxidative stress, and disorders of glucose and lipid metabolism. The active components of a single herb typically target only 1–2 of these pathological pathways. In contrast, the 17 herbs in JLD exert effects on multiple pathophysiological pathways through their respective bioactive components—for instance, ginsenoside Rg2 inhibits renal inflammation via targeting the NF-κB pathway, while total saponins from *Cornus officinalis* regulate the TGF-β1/Smad pathway to alleviate fibrosis (29, 30). This achieves a distinctive therapeutic advantage characterized by “multiple components targeting multiple pathways.” The specific discussion is as follows.

#### 4.1.1 Inhibition of inflammation and pyroptosis pathways

Inflammatory infiltration is a key driver of DN progression. Consistent with the “multiple components targeting multiple pathways” feature of JLD, the anti-inflammatory effect of the compound is achieved through coordinated regulation of multiple active ingredients on the NF-κB-related signaling cascade: ginsenoside Rg2 in *Panax ginseng* reduces excessive reactive oxygen species production, thereby inhibiting NF-κB/NLRP3 signaling pathway activation and blocking the inflammatory cascade and pyroptosis (29); brachangobinan A, a lignan from *Perilla frutescens*, directly inhibits NF-κB pathway activation and reduces pro-inflammatory factors (31); *Pueraria root* extract PTY-2r normalizes inflammatory factor and inducible nitric oxide synthase expression in renal tissue by downregulating PKC-α and NF-κB pathways (32). Furthermore, *Polygonatum* polysaccharide modulates inflammatory factor transcription by promoting IκB-α degradation and NF-κB p65 nuclear translocation, further reinforcing the compound’s anti-inflammatory network (33). This multi-component targeted regulation of inflammation lays the foundation for subsequent attenuation of DN progression.

#### 4.1.2 Anti-renal fibrosis pathway regulation

Renal fibrosis represents the core pathological alteration in end-stage DN. JLD targets key fibrosis-related pathways such as the TGF-β/Smad through multiple active components: cycloartane-type triterpenoid glycosides and aminoguanidine in *Cornus officinalis* reduce serum TGF-β1 protein and glomerular TGF-β1 mRNA expression, decreasing abnormal deposition of fibronectin and laminin in renal tissue (30); *Sophora flavescens* inhibits the activation of TGF-β/Smad pathway to reduce extracellular matrix-associated protein accumulation (34); saponins from *Semen Litchi* lower TGF-β1 and fibronectin levels while suppressing proliferation of human glomerular mesangial cells (35); 2,3,5,4’-tetrahydroxystilbene-2-O-β-D-glucoside in *Polygonum multiflorum* extract reduces VEGF expression in renal tissue, alleviates oxidative stress damage in renal vascular endothelium and proteinuria, mitigates glomerulosclerosis and interstitial fibrosis, and delays the progression of renal insufficiency (36). Collectively, these components form a multi-component anti-fibrotic regulatory network to attenuate the progression of DN to end-stage renal disease.

#### 4.1.3 Oxidative stress and podocyte protection

Oxidative stress-induced podocyte injury is a major contributor to DN proteinuria. *Rehmannia glutinosa* water extract alleviates renal pathological damage in DN model rats by enhancing antioxidant capacity and reducing inflammatory factor levels (37); meanwhile, *Poria cocos* and its outer layer components enhance antioxidant defense systems by regulating the Keap1/Nrf2 pathway (38); specifically, Tanshinone IIA targets the LAVL1-ACSL4 axis to mitigate high-glucose-induced damage and ferroptosis in MPC5 podocytes, collectively safeguarding podocyte integrity and renal function (39). Protection of podocytes and attenuation of oxidative stress are critical for reducing proteinuria and preserving renal function in DN.

#### 4.1.4 Glucose-lipid metabolism and AGE inhibition

Metabolic disorders serve as the initiating factor for DN. Polysaccharides from *Coptis chinensis* improve pancreatic and hepatic morphological abnormalities, elevate serum insulin levels, reduce FBG and glycated serum protein, inhibit AGE accumulation, and concurrently enhance renal function (40); meanwhile, Timosaponin B-II from *Anemarrhena asphodeloides* exerts dual hypoglycemic and anti-inflammatory effects by regulating the TXNIP, mTOR, and NF-κB pathways, thereby ameliorating alloxan-induced DN (41); furthermore, extracts from *Lycium chinense* indirectly alleviate renal metabolic burden by reducing hepatic lipid accumulation and white adipose tissue mass, improving adipocyte hypertrophy (42). Collectively, these components act in a coordinated manner to correct metabolic disorders and block AGE-mediated renal damage, reinforcing the multi-target therapeutic advantage of JLD.

#### 4.1.5 Cell signaling pathways and inhibition of EMT

JLD components block renal tissue epithelial-mesenchymal transition (EMT) and cellular injury by regulating key signaling pathways: Icariin from *Epimedium brevicornu* inhibits the PI3K/AKT pathway, blocking high-glucose-induced EMT in HK-2 human renal tubular epithelial cells (43); specifically, extracts from *Ophiopogon japonicus* mitigate renal tissue damage in DN model rats by modulating the EGFR/PI3K/AKT pathway (44); additionally, *Atractylodes macrocephala* directly improves renal function in DN patients through its anti-inflammatory and antioxidant properties, which in turn indirectly suppress renal epithelial cell EMT progression (45). Collectively, these components form a multi-component regulatory network targeting diverse signaling pathways, further reinforcing JLD’s multi-target therapeutic efficacy against DN progression.

### 4.2 The potential safety of JLD

Protein-bound uremic toxins (PBUTs), such as indoxyl sulfate and p-cresol sulfate, are key biomarkers of renal impairment; their elevation directly reflects impaired renal metabolic clearance and increases the risk of drug-induced adverse reactions by inducing oxidative stress, inflammation, and gut microbiota dysbiosis (46). All 13 included studies confirmed that JLD significantly improved renal function in DN patients, as evidenced by reduced SCr, BUN, and 24h-UTP. This suggests JLD may enhance renal filtration, thereby increasing PBUT clearance and reducing adverse reaction risks. However, none of the studies directly measured PBUT levels, precluding a quantitative association with adverse reaction incidence. Future studies should include PBUT dynamic monitoring and gut microbiota testing to explore whether JLD reduces PBUT production via microbiota modulation, providing direct evidence for its safety advantages.

Beyond this potential safety benefit, two critical safety concerns regarding JLD warrant in-depth discussion. First, JLD is a complex formulation of 17 herbal components each containing multiple bioactive substances, and thus inherently carries the risk of both pharmacokinetic (PK) and pharmacodynamic (PD) drug interactions. From a pharmacokinetic perspective, key bioactive components in JLD such as tanshinone IIA from *Salvia miltiorrhiza* and astragaloside IV from *Astragalus membranaceus* are well documented to modulate cytochrome P450 enzyme subtypes including CYP3A4 and CYP2C9 (47). These enzymes play a pivotal role in the metabolic clearance of first-line medications for DN, including ACEI/ARB inhibitors, SGLT2 inhibitors and sulfonylureas. Enzyme modulation by JLD components may therefore lead to either abnormal accumulation of co-administered drugs and heightened toxicity risk or accelerated drug clearance and diminished therapeutic efficacy (48). From a pharmacodynamic perspective, the sporadic occurrences of hypoglycemia and orthostatic hypotension recorded in clinical safety data likely represent direct manifestations of pharmacodynamic interactions between JLD and conventional hypoglycemic or hypotensive agents, representing direct manifestations of pharmacodynamic interactions (49). Unfortunately, the RCTs included in this meta-analysis lacked systematic reporting of detailed combination medication regimens and did not implement targeted monitoring for drug interactions, which precludes quantitative assessment of the incidence and severity of these risks (50).

Second, JLD’s acute, subacute, and chronic toxicity profiles remain unclear. While short-term data indicate minimal side effects, no published literature or systematic experimental data on JLD’s toxicity as an integrated compound exists to date. Current safety evidence is limited to short-term clinical observations, lacking 6-month to 2-year animal or human long-term toxicity evaluations to assess organ damage from component accumulation. In the absence of direct toxicological data for JLD, potential long-term risks can only be inferred from its individual herbal components: *Panax ginseng* has low acute toxicity, but long-term high-dose use may induce mild liver enzyme abnormalities (51); *Rehmannia glutinosa* shows no acute, subacute or long-term organ toxicity (52); Cornus officinalis Sieb. et Zucc has favorable long-term safety, with no liver, kidney or heart pathological changes (53); *Polyporia cocos* has no detectable acute or chronic toxicity, and long-term use does not impair gastrointestinal or renal function (38); *Eupatorium fortunei* may cause mild reversible abdominal distension with long-term high doses, and while short-term ingestion is unlikely to trigger acute toxicity, caution is still advised for prolonged administration (54); *Coptis chinensis* has low inherent toxicity but may disrupt intestinal flora in susceptible individuals with prolonged use (55); Anemarrhena asphodeloides has no reported long-term toxicity (56); *Salvia miltiorrhiza* is generally safe for long-term use, but requires liver function monitoring in pre-existing hepatic impairment patients (57); *Pueraria thomsonii* shows no significant long-term toxicity and does not affect glucose or lipid homeostasis (58); *Cortex Lycii* has low long-term risk, with mild electrolyte disturbances only in cases of excessive intake (59); *Polygonatum sibiricum* is safe chronically even at high doses (60); *Sophora flavescens* may cause mild reversible renal tubular irritation with long-term high doses (61); *Ophiopogon japonicus* has no reported long-term toxicity, supporting safety in renal disease patients (62); *Semen Litchi* is well-tolerated long-term, with only occasional mild dry mouth at high doses (63); *Polygonum multiflorum* has well-documented long-term hepatotoxicity linked to excessive THSG intake (64); *Herba epimedii* may induce mild hormonal imbalance with prolonged high-dose use, especially in endocrine disorder patients (65). Notably, the toxicity of herbal compounds cannot be equated to the sum of their individual components. TCM’s “monarch-minister-assistant-guide” compatibility principle may mitigate single-herb toxicity via component interactions, yet this hypothesis lacks validation due to insufficient dedicated toxicological studies on JLD itself.

In summary, JLD is relatively safe for short-term clinical use, but its long-term safety and drug interaction risks require further clarification. Clinically, JLD should be used under close monitoring: avoid co-administration with strong CYP450 enzyme inhibitors or inducers, regularly monitor blood glucose, blood pressure, and liver or kidney function during long-term use, and initiate treatment at a low dose and adjust according to patient tolerance. Future research priorities include (1): conducting systematic PK/PD interaction studies between JLD and common DN medications to clarify interaction mechanisms and establish safe co-administration protocols; (2) performing comprehensive toxicological evaluations of JLD (including acute, subacute, and chronic toxicity tests in animals) to determine its safe dose range and potential long-term toxic targets, thereby supporting its clinical translation. Furthermore, JLD should only be used under the guidance of a licensed physician or certified TCM practitioner.

### 4.3 Limitations of this study

Despite employing rigorous analytical methods, this study has several limitations. First, existing research on JLD has primarily focused on Eastern populations, and its efficacy and safety in Western populations remain unclear, necessitating further validation through subsequent studies. Second, the methodological quality of the included studies varied considerably. Some studies provided unclear descriptions of their randomization methods, and significant heterogeneity existed between studies. This heterogeneity may be related to differences in baseline characteristics such as age distribution, treatment duration, dosage, and disease severity among study participants. Additionally, the sample size of this study was relatively limited, and some included studies had low methodological quality, potentially introducing bias. Finally, none of the included studies reported key renal function indicators such as the urine albumin-to-creatinine ratio and estimated glomerular filtration rate. This precludes strict adherence to the “dual-track” stratification strategy recommended by the KDIGO guidelines—a strategy critical for reducing baseline population heterogeneity and enhancing the reliability of subgroup analyses. Therefore, when conducting future RCTs evaluating JLD for DN, it is recommended to strictly adhere to KDIGO stratification criteria in study design to provide more reliable evidence-based medical evidence for clinical practice.

## 5 Conclusions

The results of this study indicate that JLD, as an adjunctive therapy for DN, demonstrates certain efficacy in improving clinical rates, SCr levels, BUN levels, and 24h-UTP. However, regarding safety, no statistically significant difference in the risk of adverse reactions was observed between the JLD group and the control group, and no superior safety profile for JLD was identified.

## Data Availability

The original contributions presented in the study are included in the article/**Supplementary Material**. Further inquiries can be directed to the corresponding authors.
