# Supplementary material for: Efficacy and safety of Jinlida granules as an adjuvant treatment for diabetic nephropathy: a systematic review and meta-analysis
Source: Front Endocrinol (Lausanne). 2026 Feb 3;17:1740623. doi: 10.3389/fendo.2026.1740623 (PMC12946738; doi:10.3389/fendo.2026.1740623)
Supplement: Supplementary file 1 [file DataSheet1.docx]

Supplementary information

Efficacy and Safety of Jinlida Granules as an Adjuvant Treatment for Diabetic Nephropathy : A Systematic Review and Meta-analysis

Bo Dai ^1^, Yanxu Chen ^2^, Yang Xiao ^1^, Jinying Chen ^1^, Zexin Zhu ^1^, Peng Zhang ^1^, Jieyu Zhang ^1^, Jian Sun ^1^, Pengjie Bao ^1, 3^,Zheng Nan ^1, 3*^, Qi Zhang ^4*^

Correspondence to: Zheng Nan; [nanzheng001@aliyun.com](mailto:nanzheng001@aliyun.com); Qi Zhang 596058757@qq.com

| Drug composition of JLD | Drug grams | Drug ratio |
| --- | --- | --- |
| Ginseng | 184.5 | 6.63 |
| Rhizoma Polygonati | 244.5 | 8.79 |
| Atractylodes Macrocephala Koidz. (stir-fried with bran) | 122.2 | 4.39 |
| Sophora Flavescens Root | 100 | 3.59 |
| Ophiopogon Japonicus (Thunb.) Ker-Gawl. | 244.5 | 8.79 |
| Rehmannia Glutinosa Libosch. | 184.5 | 6.63 |
| Prepared Polygonum Multiflorum Thunb. | 149 | 5.35 |
| Cornus Officinalis Sieb. et Zucc. | 244.5 | 8.79 |
| Poria Cocos (Schw.) Wolf | 149 | 5.35 |
| Eupatorium Fortunei Turcz. | 100 | 3.59 |
| Coptis Chinensis Franch. | 100 | 3.59 |
| Anemarrhena Asphodeloides Bunge | 122.2 | 4.39 |
| Epimedium Brevicornu Maxim. (roasted) | 100 | 3.59 |
| Salvia Miltiorrhiza Bunge | 160 | 5.75 |
| Pueraria Lobata (Willd.) Ohwi | 244.5 | 8.79 |
| Semen Litchi | 244.5 | 8.79 |
| Cortex Lycii Radicis | 149 | 5.35 |

**Supplementary Material S1(Ratio of the ingredients of the Jinlida granules)**

**Supplementary Material S2 (Preparation method)**

The above seventeen ingredients are processed as follows: The volatile oils are extracted from *Pogostemon cablin (Blanco) Cass.* and bran-fried *Atractylodes lancea (Thunb.) DC*. by steam distillation. The resulting aqueous distillate is filtered and reserved. *Cornus officinalis Siebold & Zucc.* is macerated for 24 hours with seven times the amount of 75% ethanol as the solvent, followed by percolation. The percolate is collected, the ethanol is recovered, and the solution is concentrated to a thick extract with a relative density of 1.30–1.35 (at 60°C), which is then dried and reserved.

*Panax ginseng C.A.Mey.*, *Ophiopogon japonicus (L.f.) Ker Gawl.*, *Epimedium brevicornu Maxim.* (processed with honey), *Anemarrhena asphodeloides Bunge*, and *Pueraria montana (Lour.) Merr*. (powdered form) are subjected to reflux extraction with ethanol three times, each lasting two hours. The extracts are combined, filtered, and the ethanol is recovered. The filtrate is concentrated to a thick extract with a relative density of 1.30–1.35 (at 60°C), which is then dried and reserved.

The remaining nine ingredients, including *Polygonatum kingianum Collett & Hemsl.*, are decocted twice with water, each time for two hours. The decoctions are filtered, combined, and then merged with the previously reserved aqueous distillate. The mixture is concentrated to a clear extract with a relative density of 1.10–1.15 (at 60°C). Ethanol is added to achieve an alcohol content of 60%, and the solution is refrigerated for 24 hours. After filtration, the ethanol is recovered, and the filtrate is concentrated to a thick extract with a relative density of 1.30–1.35 (at 60°C), which is then dried.

All the dried extracts mentioned above are combined, pulverized into a fine powder, and mixed with an appropriate amount of lactose powder and dextrin. The mixture is granulated, dried, and then the volatile oil is sprayed in and uniformly blended. The final product is prepared to a total weight of 1000 g.

# Supplementary information S3 (PRISMA 2020 checklist)

| **Section and Topic** | **Item #** | **Checklist item** | **Location where item**  **is reported** |
| --- | --- | --- | --- |
| **TITLE** | | |  |
| Title | 1 | Identify the report as a systematic review. | P1 |
| **ABSTRACT** | | |  |
| Abstract | 2 | See the PRISMA 2020 for Abstracts checklist. | P1-3 |
| **INTRODUCTION** | | |  |
| Rationale | 3 | Describe the rationale for the review in the context of existing knowledge. | P5-7 |
| Objectives | 4 | Provide an explicit statement of the objective(s) or question(s) the review addresses. | P6-7 |
| **METHODS** | | |  |
| Eligibility criteria | 5 | Specify the inclusion and exclusion criteria for the review and how studies were grouped for the syntheses. | P7-10 |
| Information sources | 6 | Specify all databases, registers, websites, organisations, reference lists and other sources searched or consulted to identify studies. Specify the date when each source was last searched or consulted. | P7 |
| Search strategy | 7 | Present the full search strategies for all databases, registers and websites, including any filters and limits used. | P10 |
| Selection process | 8 | Specify the methods used to decide whether a study met the inclusion criteria of the review, including how many reviewers screened each record and each report retrieved, whether they worked independently, and if applicable, details of automation tools used in the process. | P10 |
| Data collection process | 9 | Specify the methods used to collect data from reports, including how many reviewers collected data from each report, whether they worked independently, any processes for obtaining or confirming data from study investigators, and if applicable, details of automation tools used in the process. | P10 |
| Data items | 10a | List and define all outcomes for which data were sought. Specify whether all results that were compatible with each outcome domain in each study were sought (e.g. for all measures, time points, analyses), and if not, the methods used to decide which results to collect. | P10 |
|  | 10b | List and define all other variables for which data were sought (e.g. participant and intervention characteristics, funding sources). Describe any assumptions made about any missing or unclear information. | P6-7 |
| Study risk of bias assessment | 11 | Specify the methods used to assess risk of bias in the included studies, including details of the tool(s) used, how many reviewers assessed each study and whether they worked independently, and if applicable, details of automation tools used in the process. | P10 |
| Effect measures | 12 | Specify for each outcome the effect measure(s) (e.g. risk ratio, mean difference) used in the synthesis or presentation of results. | P10 |
| Synthesis methods | 13a | Describe the processes used to decide which studies were eligible for each synthesis (e.g. tabulating the study intervention characteristics and comparing against the planned groups for each synthesis (item #5)). | P10-11 |
|  | 13b | Describe any methods required to prepare the data for presentation or synthesis, such as handling of missing summary statistics, or data conversions. | P10-11 |
|  | 13c | Describe any methods used to tabulate or visually display results of individual studies and syntheses. | P10-11 |
|  | 13d | Describe any methods used to synthesize results and provide a rationale for the choice(s). If meta-analysis was performed, describe the model(s), method(s) to identify the presence and extent of statistical heterogeneity, and software package(s) used. | P10-11 |
|  | 13e | Describe any methods used to explore possible causes of heterogeneity among study results (e.g. subgroup analysis, meta-regression). | P10-11 |
|  | 13f | Describe any sensitivity analyses conducted to assess robustness of the synthesized results. | P10-11 |
| Reporting bias assessment | 14 | Describe any methods used to assess risk of bias due to missing results in a synthesis (arising from reporting biases). | P10-11 |


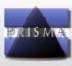
**PRISMA 2020 Checklist**

| **Section and Topic** | **Item #** | **Checklist item** | **Location where item**  **is reported** |
| --- | --- | --- | --- |
| Certainty assessment | 15 | Describe any methods used to assess certainty (or confidence) in the body of evidence for an outcome. | P10-11 |
| **RESULTS** | | |  |
| Study selection | 16a | Describe the results of the search and selection process, from the number of records identified in the search to the number of studies included in the review, ideally using a flow diagram. | P11, Figure 1 |
|  | 16b | Cite studies that might appear to meet the inclusion criteria, but which were excluded, and explain why they were excluded. | P11, Figure 1 |
| Study characteristics | 17 | Cite each included study and present its characteristics. | P12, Table2 |
| Risk of bias in studies | 18 | Present assessments of risk of bias for each included study. | P12, Figure 2 |
| Results of individual studies | 19 | For all outcomes, present, for each study: (a) summary statistics for each group (where appropriate) and (b) an effect estimate and its precision (e.g. confidence/credible interval), ideally using structured tables or plots. | P12-14,  Table3,Supplementary information S14-16 |
| Results of syntheses | 20a | For each synthesis, briefly summarise the characteristics and risk of bias among contributing studies. | / |
|  | 20b | Present results of all statistical syntheses conducted. If meta-analysis was done, present for each the summary estimate and its precision (e.g. confidence/credible interval) and measures of statistical heterogeneity. If comparing groups, describe the direction of the effect. | P12-14,  Table3,Supplementary information S14-16 |
|  | 20c | Present results of all investigations of possible causes of heterogeneity among study results. | P14 |
|  | 20d | Present results of all sensitivity analyses conducted to assess the robustness of the synthesized results. | / |
| Reporting biases | 21 | Present assessments of risk of bias due to missing results (arising from reporting biases) for each synthesis assessed. | P14 |
| Certainty of evidence | 22 | Present assessments of certainty (or confidence) in the body of evidence for each outcome assessed. | P14 |
| **DISCUSSION** | | |  |
| Discussion | 23a | Provide a general interpretation of the results in the context of other evidence. | P15-22 |
|  | 23b | Discuss any limitations of the evidence included in the review. | P21-22 |
|  | 23c | Discuss any limitations of the review processes used. | P21-22 |
|  | 23d | Discuss implications of the results for practice, policy, and future research. | P15-21 |
| **OTHER INFORMATION** | | |  |
| Registration and protocol | 24a | Provide registration information for the review, including register name and registration number, or state that the review was not registered. | P7,CRD420251148725 |
|  | 24b | Indicate where the review protocol can be accessed, or state that a protocol was not prepared. | P7,CRD420251148725 |
|  | 24c | Describe and explain any amendments to information provided at registration or in the protocol. | / |
| Support | 25 | Describe sources of financial or non-financial support for the review, and the role of the funders or sponsors in the review. | P22 |
| Competing interests | 26 | Declare any competing interests of review authors. | P22 |
| Availability of data, code and other informations | 27 | Report which of the following are publicly available and where they can be found: template data collection forms; data extracted from included studies; data used for all analyses; analytic code; any other informations used in the review. | P22 |

*From:* Page MJ, McKenzie JE, Bossuyt PM, et al. The PRISMA 2020 statement: an updated guideline for reporting systematic reviews. *BMJ* 2021;372:n71. doi:10.1136/bmj.n71

For more information, visit: **w ww.prisma-statement.org**

**Supplementary Material S4 (Other Database Retrieval Strategies)**

PubMed

((("Diabetic Nephropathies"[Mesh]) OR (((((((((((((((((Nephropathies, Diabetic) OR (Nephropathy, Diabetic)) OR (Diabetic Nephropathy)) OR (Diabetic Kidney Disease)) OR (Diabetic Kidney Diseases)) OR (Kidney Disease, Diabetic)) OR (Kidney Diseases, Diabetic)) OR (Diabetic Glomerulosclerosis)) OR (Glomerulosclerosis, Diabetic)) OR (Intracapillary Glomerulosclerosis)) OR (Nodular Glomerulosclerosis)) OR (Glomerulosclerosis, Nodular)) OR (Kimmelstiel-Wilson Syndrome)) OR (Kimmelstiel Wilson Syndrome)) OR (Syndrome, Kimmelstiel-Wilson)) OR (Kimmelstiel-Wilson Disease)) OR (Kimmelstiel Wilson Disease))) AND ((Jinlida granules) OR (Jinlida))) AND (randomized controlled trial[Publication Type] OR randomized[Title/Abstract] OR placebo[Title/Abstract] RCT)

Embase

'Nephropathies, Diabetic':ab,ti OR 'Nephropathy, Diabetic':ab,ti OR 'Diabetic Nephropathy':ab,ti OR 'Diabetic Kidney Disease':ab,ti OR 'Diabetic Kidney Diseases':ab,ti OR 'Kidney Disease, Diabetic':ab,ti OR 'Kidney Diseases, Diabetic':ab,ti OR 'Diabetic Glomerulosclerosis':ab,ti OR 'Glomerulosclerosis, Diabetic':ab,ti OR 'Intracapillary Glomerulosclerosis':ab,ti OR 'Nodular Glomerulosclerosis':ab,ti OR 'Glomerulosclerosis, Nodular':ab,ti OR 'Kimmelstiel-Wilson Syndrome':ab,ti OR 'Kimmelstiel Wilson Syndrome':ab,ti OR 'Syndrome, Kimmelstiel-Wilson':ab,ti OR 'Kimmelstiel-Wilson Disease':ab,ti OR 'Kimmelstiel Wilson Disease':ab,ti

'Jinlida granule':ab,ti OR 'Jinlida':ab,ti

'randomized controlled trial':ab,ti OR 'randomized':ab,ti OR 'placebo':ab,ti

Web of Science

#1 Diabetic Nephropathies OR Nephropathies, Diabetic OR Nephropathy, Diabetic OR Diabetic Nephropathy OR Diabetic Kidney Disease OR Diabetic Kidney Diseases OR Kidney Disease, Diabetic OR Kidney Diseases, Diabetic OR Diabetic Glomerulosclerosis OR Glomerulosclerosis, Diabetic OR Intracapillary Glomerulosclerosis OR Nodular Glomerulosclerosis OR Glomerulosclerosis, Nodular OR Kimmelstiel-Wilson Syndrome OR Kimmelstiel Wilson Syndrome OR Syndrome, Kimmelstiel-Wilson OR Kimmelstiel-Wilson Disease OR Kimmelstiel Wilson Disease

#2 Jinlida granules OR Jinlida granule OR Jinlida

#3 randomized controlled trial OR randomized OR placebo RCT

#4 #1 AND #2 AND #3

The Cochrane Library

#1 Diabetic Nephropathies

#2 (Nephropathies, Diabetic):ab,ti,kw OR (Nephropathy, Diabetic):ab,ti,kw OR (Diabetic Nephropathy):ab,ti,kw OR (Diabetic Kidney Disease):ab,ti,kw OR (Diabetic Kidney Diseases):ab,ti,kw OR (Kidney Disease, Diabetic):ab,ti,kw OR (Kidney Diseases, Diabetic):ab,ti,kw OR (Diabetic Glomerulosclerosis):ab,ti,kw OR (Glomerulosclerosis, Diabetic):ab,ti,kw OR (Intracapillary Glomerulosclerosis):ab,ti,kw OR (Nodular Glomerulosclerosis):ab,ti,kw OR (Glomerulosclerosis, Nodular):ab,ti,kw OR (Kimmelstiel-Wilson Syndrome):ab,ti,kw OR (Kimmelstiel Wilson Syndrome):ab,ti,kw OR (Syndrome, Kimmelstiel-Wilson):ab,ti,kw OR (Kimmelstiel-Wilson Disease):ab,ti,kw OR (Kimmelstiel Wilson Disease):ab,ti,kw

#3 #1 OR #2

#4 Jinlida granules

#5 (Jinlida granule):ab,ti,kw OR (Jinlida):ab,ti,kw

#6 #4 OR #5

#7 (randomized controlled trial):ab,ti,kw OR (randomized):ab,ti,kw OR (placebo RCT):ab,ti,kw

#8 #3 AND #6 AND #7

China National Knowledge Infrastructure

（主题：糖尿病肾病）OR（主题：糖尿病肾脏病变）OR（主题：糖尿病肾脏损害）OR（主题：消渴肾病）OR（主题：早期糖尿病肾病）OR（主题：2型糖尿病肾病）OR（主题：糖尿病性肾病）OR（主题：糖尿病性肾小球硬化症）AND（主题：津力达颗粒）OR（主题：津力达）OR（主题：津力达口服液）OR（主题：津力达丸）OR（主题：津力达浓缩丸）AND（全文：随机对照试验（精确））OR（全文：随机对照实验（精确））OR（全文：随机对照研究（精确））OR（全文：随机对照研究（精确））OR（全文：随机对照（精确））OR（全文：随机（精确））

Wanfang Data

主题:(糖尿病肾病 or 糖尿病肾脏病变 or 糖尿病肾脏损害 or 消渴肾病 or 早期糖尿病肾病 or 2型糖尿病肾病 or 糖尿病性肾病 or 糖尿病性肾小球硬化症) and 主题:(津力达颗粒 or 津力达) and 全部:(随机对照试验 or 随机对照实验 or 随机对照研究 or RCT or 随机对照 or 随机)

The China Science and Technology Journal Database

主题:(糖尿病肾病 or 糖尿病肾脏病变 or 糖尿病肾脏损害 or 消渴肾病 or 早期糖尿病肾病 or 2型糖尿病肾病 or 糖尿病性肾病 or 糖尿病性肾小球硬化症) and 主题:(津力达颗粒 or 津力达 ) and 主题:(随机对照试验 or 随机对照实验 or 随机对照研究 or RCT or 随机对照 or 随机)

**Supplementary Material S5 (SCr Subgroup Analysis Forest Plot)**

**Supplementary Material S5 (SCr Subgroup Analysis Forest Plot)**

**Supplementary Material S6 (BUN Subgroup Analysis Forest Plot)**

**Supplementary Material S6 (BUN Subgroup Analysis Forest Plot)**

**Supplementary Material S7 (24-h UTP Subgroup Analysis Forest Plot)**

**Supplementary Material S7 (24-h UTP Subgroup Analysis Forest Plot)**

**Supplementary Material S8 (UAER forest chart)**


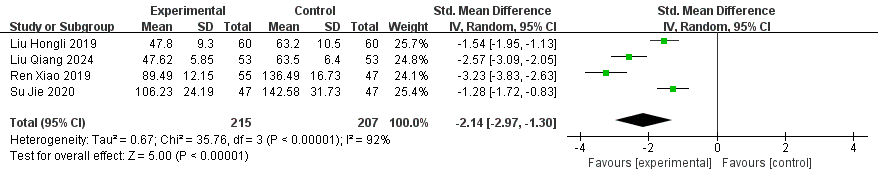


**Supplementary Material S9 (FBG forest chart)**

**
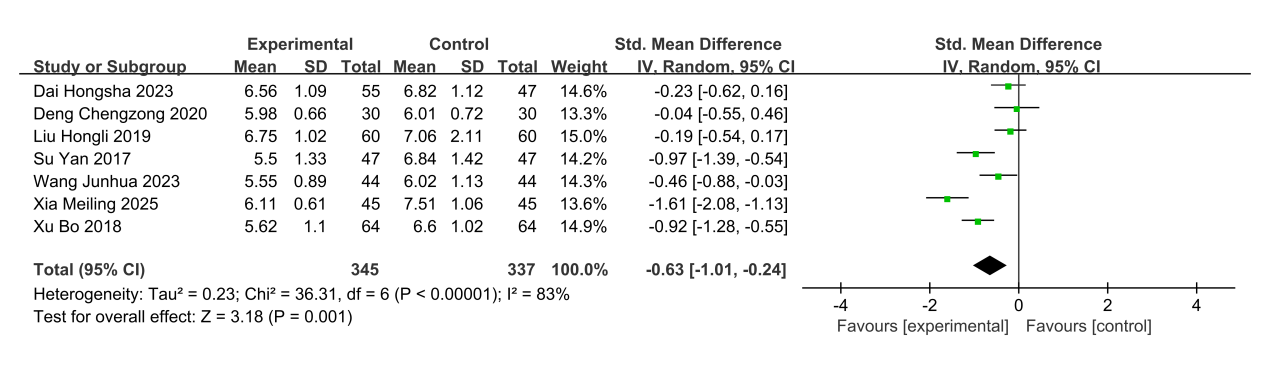
**

**Supplementary Material S10 (2hPG forest chart)**


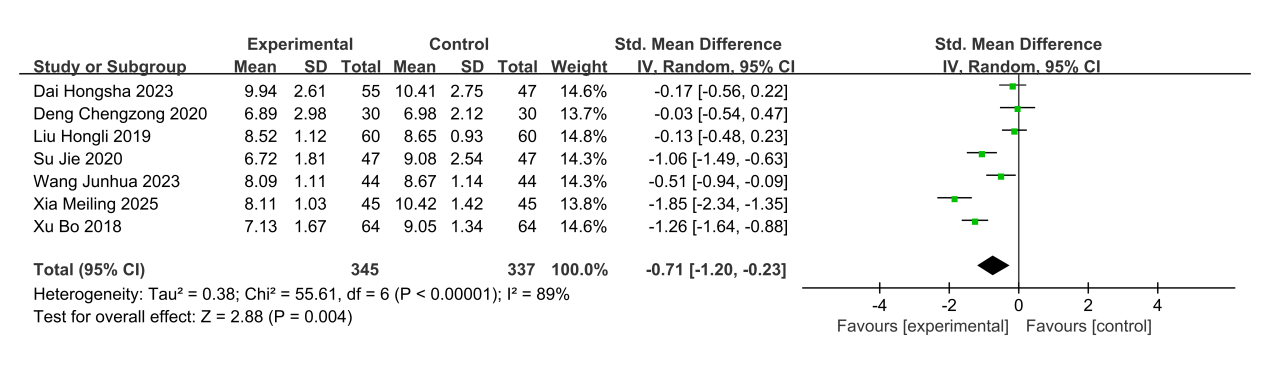


**Supplementary Material S11 (HbA1c forest chart)**

**
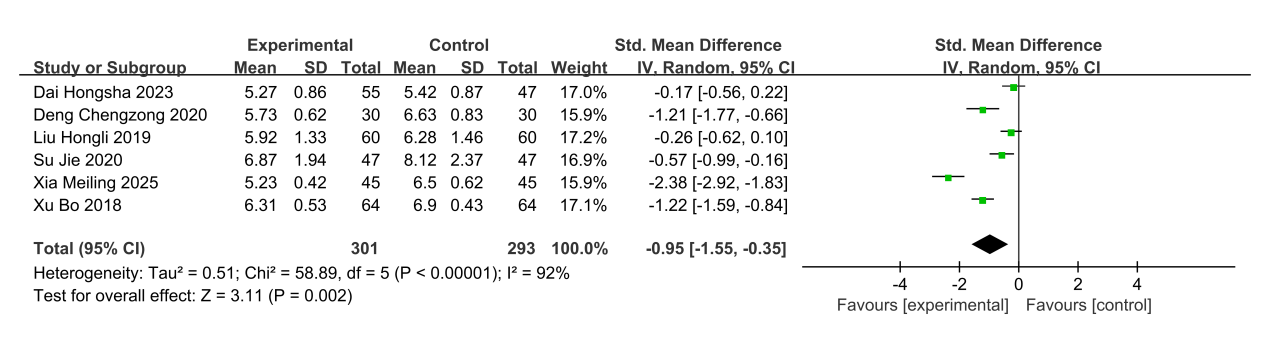
**

**Supplementary Material S12(TC forest chart)**

**
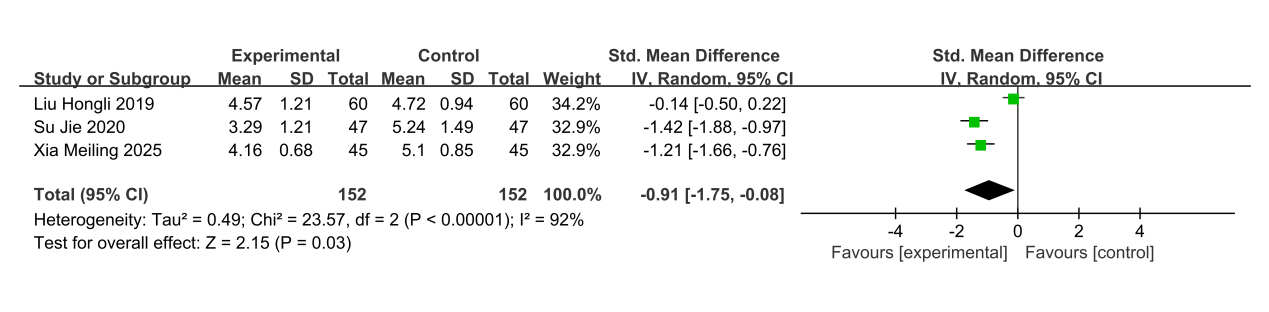
**

**Supplementary Material S13(TG forest chart）**
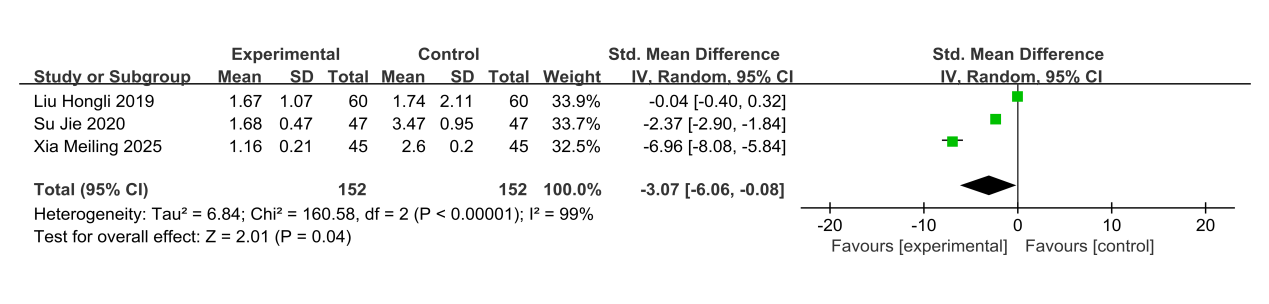


**Supplementary Material S14(IGF-1 forest chart)**

**
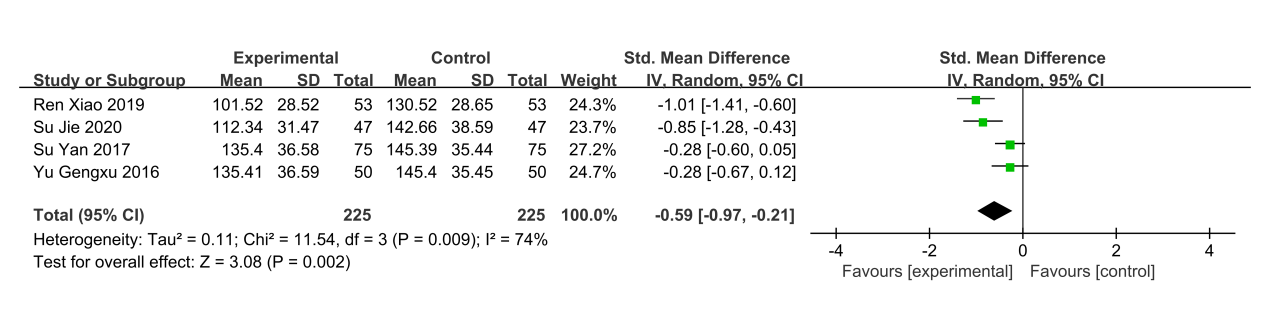
**

**Supplementary Material S15(VEGF forest chart)**

**
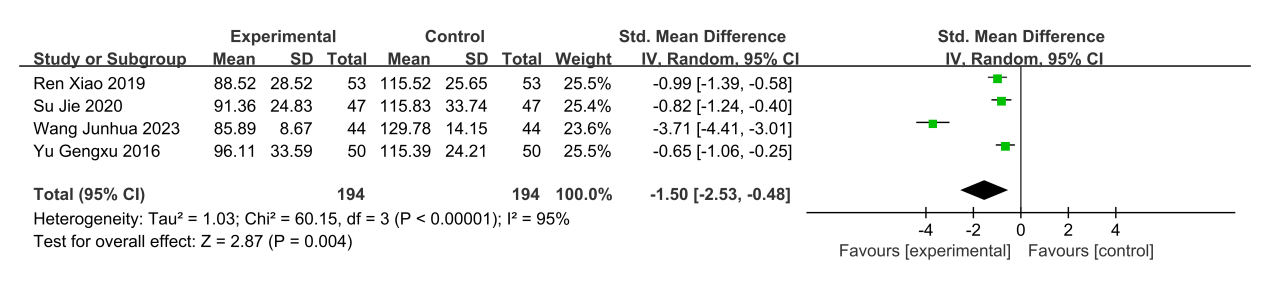
**

**Supplementary Material S16(hs-CRP forest chart)**

**
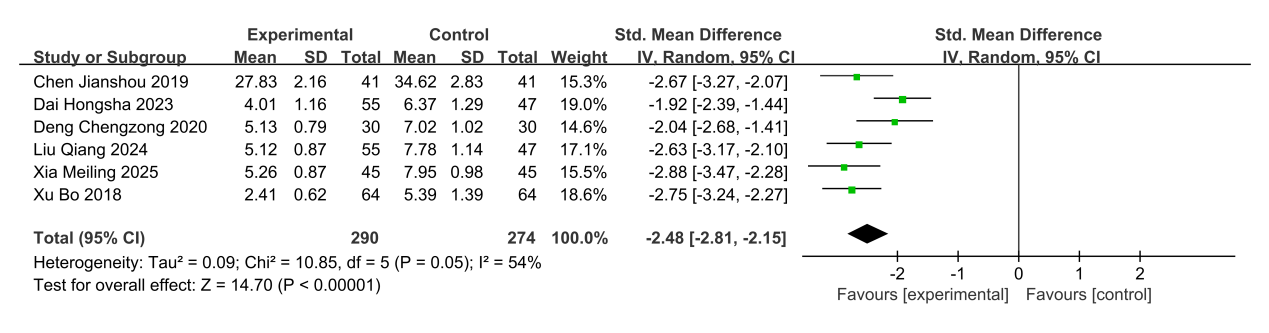
**

**Supplementary Material S17(IL-6 forest chart)**

**
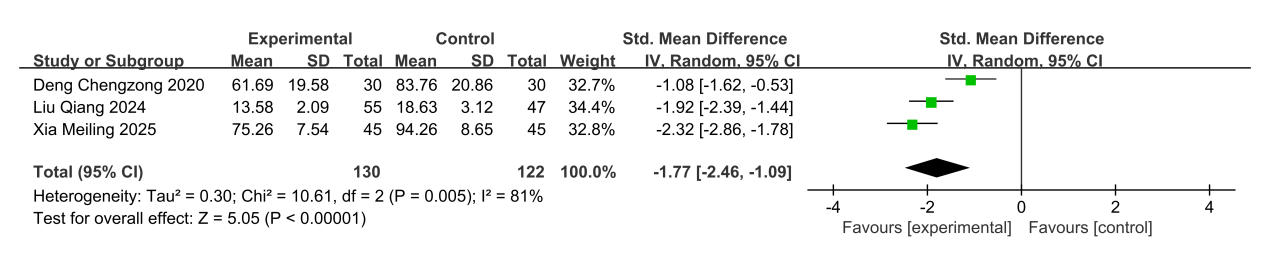
**

**Supplementary Material S18(TNF-α forest chart)**

**
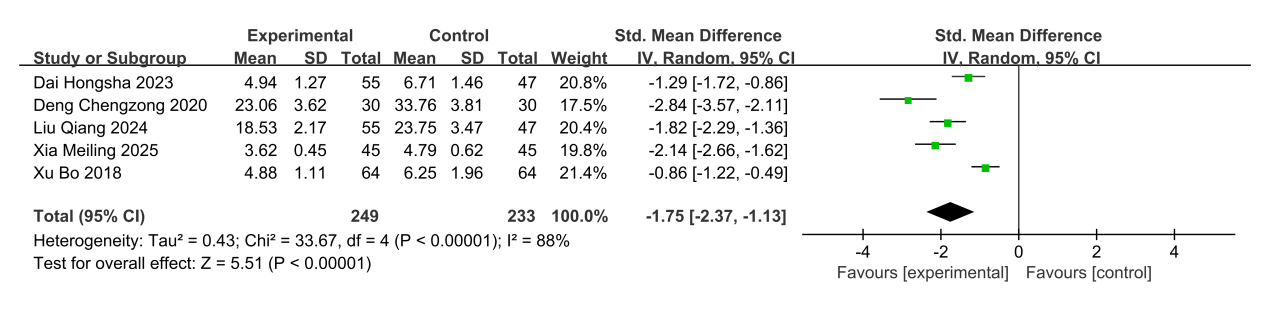
**

**Supplementary Material S19 (Adverse effect forest chart)**

**
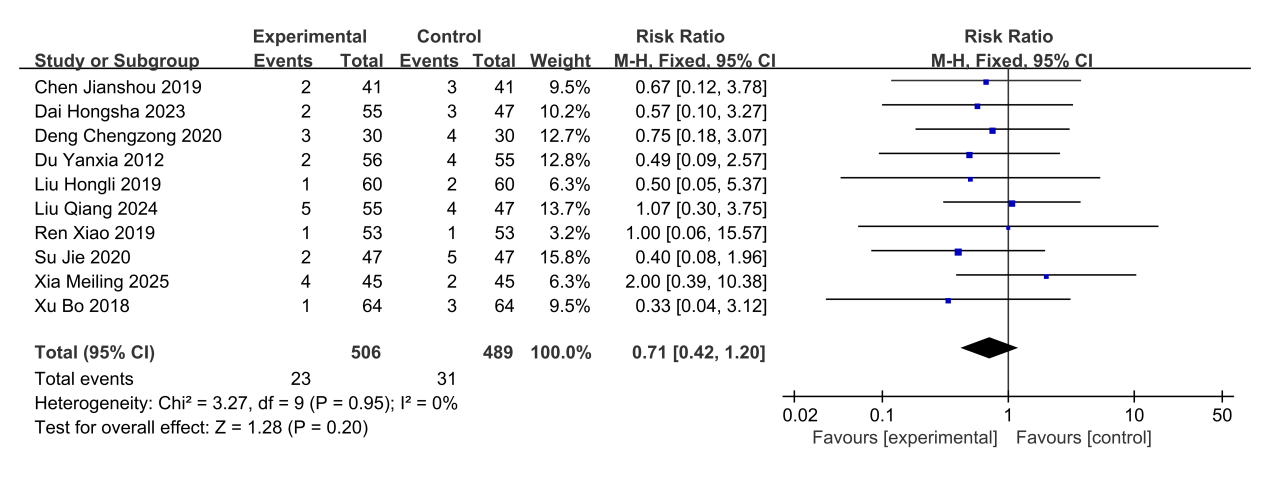
**

**Supplementary Material S20 (Scr Sensitivity Analysis Chart)**

**
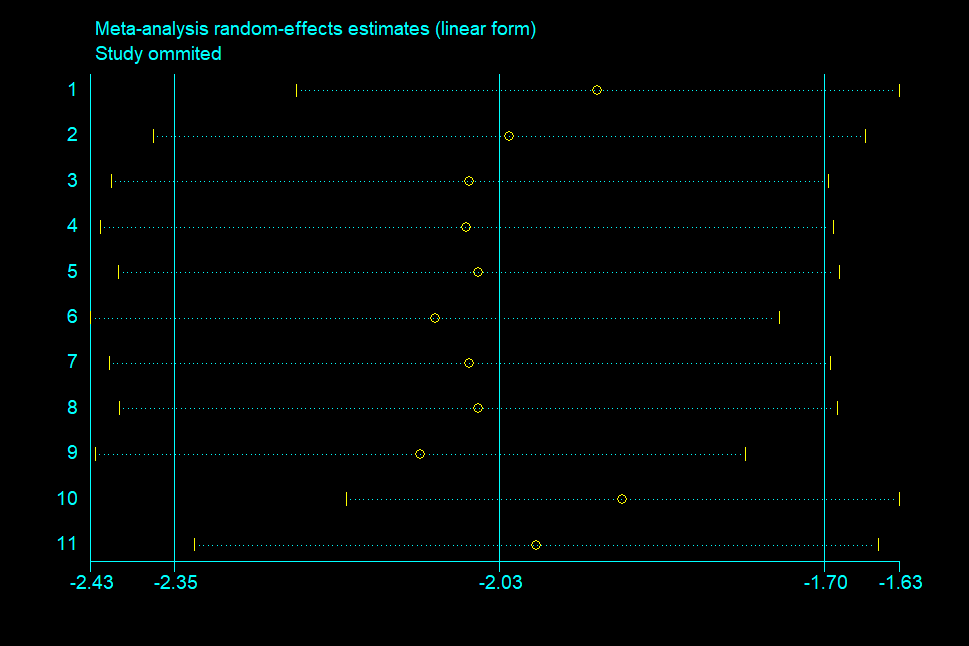
**

**Supplementary Material S21 (BUN Sensitivity Analysis Chart)**

**
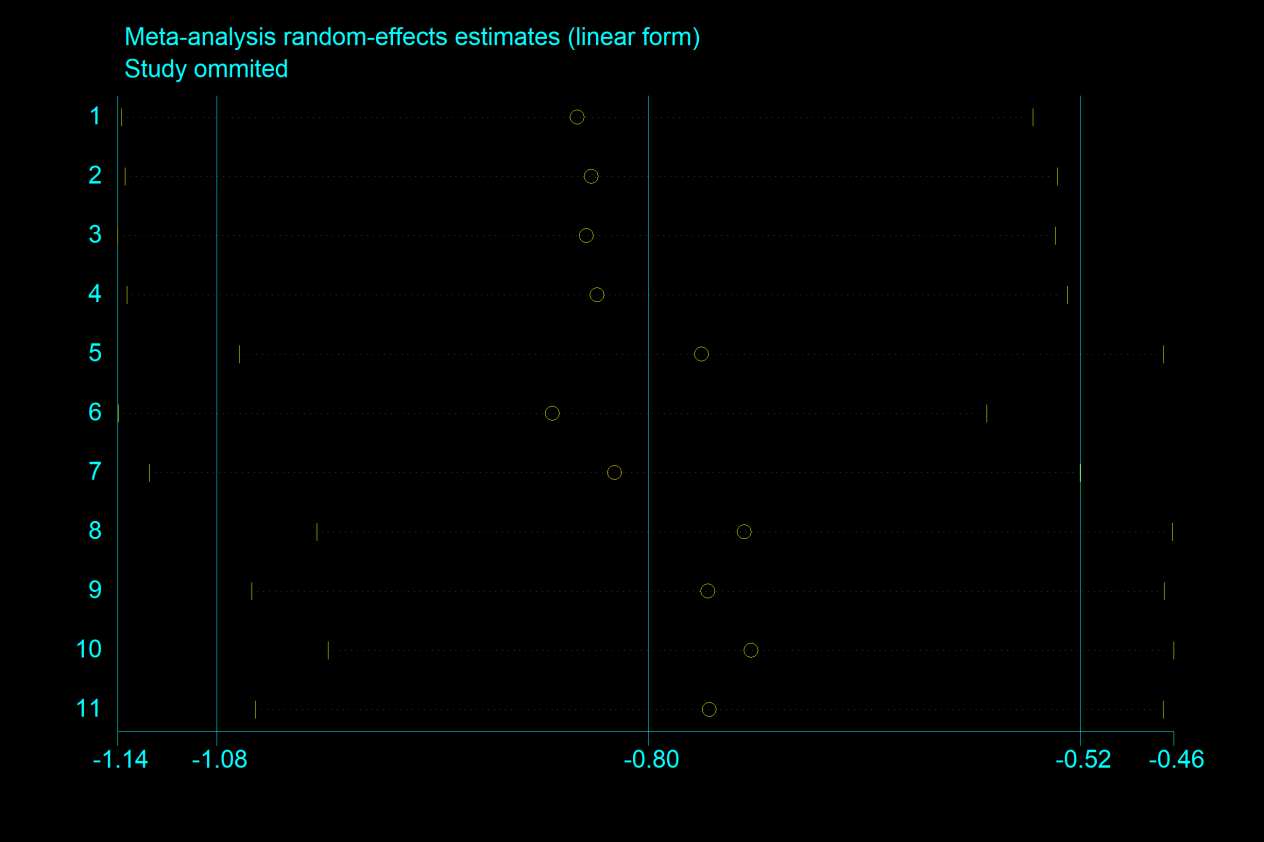
**

**Supplementary Material S22 (24h-UTP Sensitivity Analysis Chart)**

**
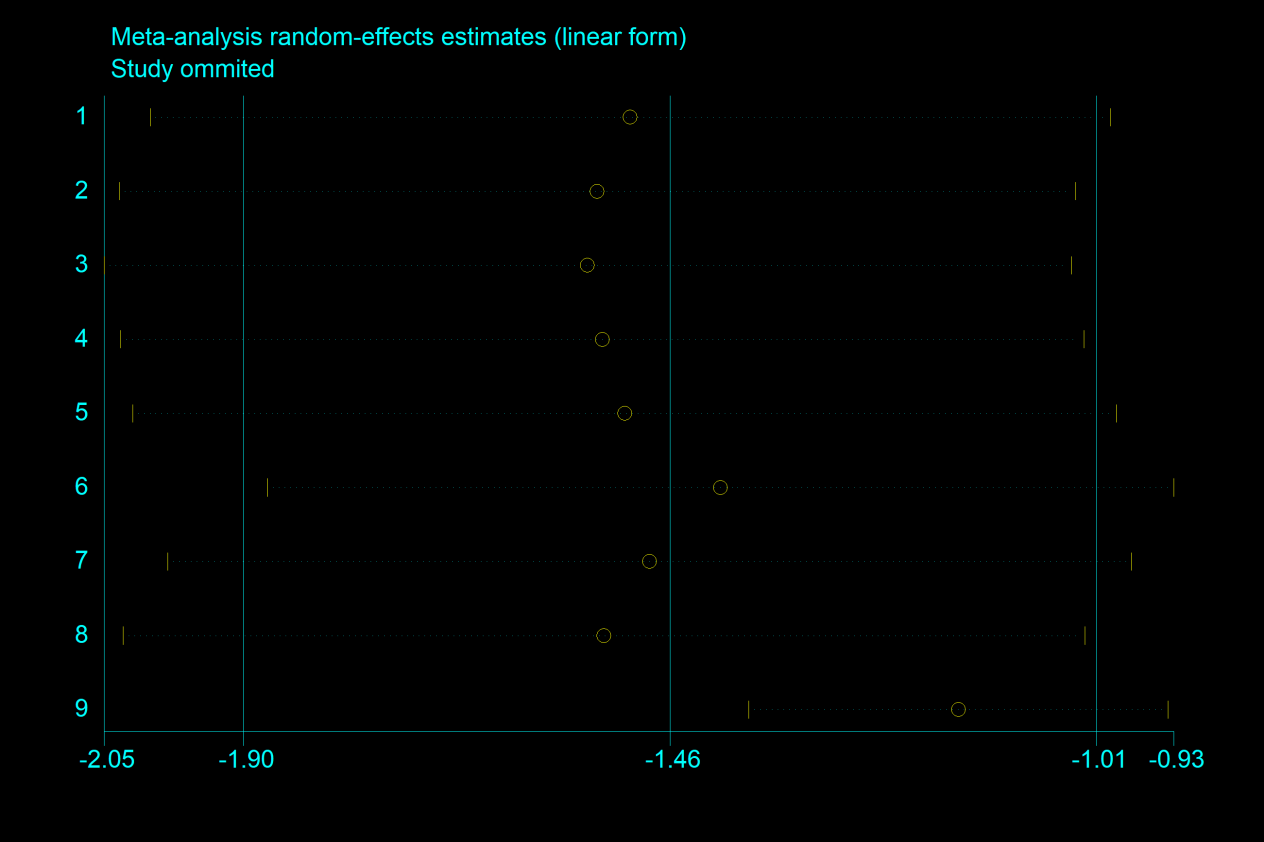
**
